# Phytochemical Analysis and Evaluation of Antioxidant and Antimicrobial Properties of Essential Oils and Seed Extracts of *Anethum graveolens* from Southern Morocco: In Vitro and In Silico Approach for a Natural Alternative to Synthetic Preservatives

**DOI:** 10.3390/ph17070862

**Published:** 2024-07-01

**Authors:** Nadia Hadi, Aziz Drioiche, El Moumen Bouchra, Soukayna Baammi, Abdelaaty Abdelaziz Shahat, Imane Tagnaout, Mohamed Radi, Fidaous Remok, Amal Bouzoubaa, Touriya Zair

**Affiliations:** 1 Research Team of Chemistry of Bioactive Molecules and the Environment, Laboratory of Innovative Materials and Biotechnology of Natural Resources, Faculty of Sciences, Moulay Ismaïl University, B.P. 11201, Zitoune, Meknes 50070, Morocco; nadia.hadi1@gmail.com (N.H.); elmoumen.bouchra4@gmail.com (E.M.B.); ema.tagnaout@gmail.com (I.T.); oncaradi@gmail.com (M.R.); remok.firdaous@gmail.com (F.R.); bouzoubaa.amal@yahoo.fr (A.B.); 2 Bioinformatics Laboratory, College of Computing, Mohammed VI Polytechnic University, Ben Guerir 43150, Morocco; soukayna.baammi@um6p.ma; 3 Pharmacognosy Department, College of Pharmacy, King Saud University, Riyadh 11451, Saudi Arabia; ashahat@ksu.edu.sa

**Keywords:** *Anethum graveolens*, GC/MS, HPLC/UV-ESI-MS, polyphenols, flavonoids, antioxidants, antimicrobials

## Abstract

*Anethum graveolens* is an aromatic plant traditionally used as an antispasmodic and carminative. The objective of this study is to analyze the chemical composition of the essential oils and extracts obtained from seeds gathered in Errachidia, southern Morocco. Additionally, the antioxidant and antimicrobial properties of these oils and extracts will be evaluated. GC-MS analysis of the EO isolated by hydrodistillation revealed that its main compounds were E-anethole (38.13%), estragole (29.32%), fenchone (17.21%), and α-pinene (7.37%). The phenolic components were extracted using the methods of decoction and Soxhlet. The assay of the phenolic compounds showed that *A. graveolens* seeds contained considerable amounts of polyphenols, flavonoids, and condensed tannins, with variable levels depending on the extract analyzed. HPLC/UV-ESI-MS analyses performed on the decoction revealed a structural diversity of the molecules present in this extract, the most important of which were umbelliferone (12.35%), 3-hydroxyflavone (11.23%), rosmanol (8.95%), biotin (8.36%), emmotin H (4.91%), and coumarin (4.21%). The antioxidant activity, as determined by three techniques (DPPH•, FRAP, and CAT), demonstrated that the essential oils (EOs) and extracts had a potent capacity to counteract detrimental free radicals, control the generation of reactive oxygen species, and mitigate oxidative damages. The antimicrobial activity of the Eos and extracts was carried out in a liquid medium against five strains (*E. cloacae*, *K. pneumoniae*, *E. coli*, *S. aureus*, and *S. epidermidis*) and four candidiasis (*C. albicans*, *C. dubliniensis*, *C. tropicalis*, and *C. parapsilosis*) and *Aspergillus niger*. The results showed the effectiveness of the EOs compared to the aqueous, ethanolic, and decoction extracts against most of the microorganisms tested. In addition, the ethanolic extract showed antifungal activity that was distinguished from that of the other extracts. The antimicrobial efficacy of the essential oils under study can primarily be attributed to the synergistic interactions among its three principal constituents (E-anethole, estragole, and fenchone). Furthermore, molecular docking and molecular dynamics simulation results reveal significant interactions and stability between the selected bioactive compounds and different target proteins involved in antimicrobial and antioxidant activities. Compounds like 3-hydroxyflavone, emmotin H, trans-caftaric acid, methyl rosmarinate, 1-caffeoyl-beta-D-glucose, and kaempferol exhibited better binding energies with the explored proteins, indicating their potential as antimicrobial and antioxidant agents. Finally, our findings emphasize the significance of *A. graveolens* seeds as a promising reservoir of advantageous health compounds that can serve as organic substitutes for the presently employed synthetic preservatives.

## 1. Introduction

Foodborne illnesses pose a significant threat to public health worldwide, contributing to substantial socioeconomic impacts, especially in developing countries [1]. The World Health Organization (WHO) has reported that antimicrobial resistance caused 1.27 million deaths globally in 2019 and contributed to 4.95 million deaths in 2023 (WHO). In response to these alarming trends, extensive research is being conducted to develop healthy food products and new antimicrobial agents.

On the other hand, the use of synthetic preservatives such as BHA and BHT to prevent food deterioration has become restricted due to their induction of DNA damage and toxicity [2,3]. From this perspective, consumers are concerned about their adverse effects on human health. This fact has led industries and consumers to search for and consume natural derivatives with antioxidant and antimicrobial properties. Among these natural substances are the essential oils and extracts from medicinal plants. These plants are considered a huge source of active secondary metabolites with medicinal properties. These compounds are primarily involved in defense mechanisms against pathogens, herbivores, and their competitors [4]. Plants have remarkably and diversely contributed to various industries such as pharmaceuticals, agriculture, cosmetics, and industrial raw materials. Indeed, they play two major roles: firstly, providing a pleasant taste to foods; secondly, preserving foods through their antimicrobial and antioxidant potentials, thereby delaying their deterioration. One of the popular plants used in pharmaceutical and agri-food industries is *Anethum graveolens*, commonly known as dill [5]. This plant is a perennial herbaceous species in the *Apiaceae* family, and it is the sole member of the *Anethum* genus. It typically reaches a height of 40 to 60 cm.

Dill, a significant plant, is produced in Eurasia and Mediterranean nations. Its seeds and leaves have been utilized as a herb or spice for flavoring dishes for 5000 years by the Egyptians and Greeks [6]. In traditional medicine, dill is generally employed for alleviating colic in infants and flatulence in young children. Additionally, its seed is used in decoction or infusion mode for the treatment of urinary infections, insomnia, hyposecretion of milk, digestive disorders accompanied by meteorism, and gastrointestinal spasms [6,7]. It is commonly employed as a natural flavor enhancer in many culinary items within the industry, particularly in sauces, salads, soups, shellfish, meats, fries, and pickles. The volatile oil derived from the aboveground portion is utilized as a taste enhancer in culinary and beverage applications owing to its agreeable fragrance. Moreover, the essential oils are employed as a scent to enhance the smell of soaps, detergents, and cosmetics [8,9].

At the scientific level, numerous previous studies have highlighted the biological potential of *Anethum graveolens*, such as anti-inflammatory [10], antidiabetic [11], antimicrobial [12], diuretic [13], insecticidal [14], hepatoprotective [15], anticancer, and antioxidant [16].

Due to its intriguing characteristics, the global economic significance of this species has greatly escalated. In Morocco, this plant holds ecological, socio-economic, and therapeutic significance. However, there is limited information on the chemical composition and biological characteristics of the extracts and essential oils derived from *Anethum graveolens* originating from Morocco. Our study specifically examined the detailed examination of the chemical compounds in the essential oil and extracts of *Anethum graveolens* seeds from the Boulemane area. Moreover, our work aimed to discover naturally occurring chemical compounds with antioxidant capabilities and assess the antioxidant and antibacterial activities of the extracts and essential oils derived from this species using in vitro testing.

Nevertheless, there has been a limited amount of research focused on investigating the chemical composition of extracts derived from *Anethum graveolens* seeds. This study was conducted within the specified context, aiming to contribute to the phytochemical analysis and characterization of chemical families found in *Anethum graveolens* seeds gathered from the Boulemane region. Additionally, it entails the assessment of the antibacterial and antioxidant properties of the essential oils and extracts by in vitro and in silico methods in order to uncover their potential as antioxidants and antimicrobials.

## 2. Results & Discussion

### 2.1. Quality Control of Plant Material

The quality control findings of the examined plant material are displayed in Table 1. The seeds of *A. graveolens* had a moisture content of around 15.80%, which was within the typical range for seeds. The plant extract had a pH that was somewhat acidic, which aligned with its mineral content of 15.03%. This pH level meets the quality criteria set by AFNOR (NF ISO 5984).

Medicinal plants, despite their health advantages, frequently become polluted by different agents or poisonous compounds during their cultivation and processing. Out of these, heavy metals are particularly worrisome since they can disrupt the proper operation of the central nervous system, liver, lungs, heart, kidneys, and brain. This disruption can result in hypertension, stomach aches, skin rashes, intestinal ulcers, and different types of cancer. In order to tackle this problem, we utilized atomic absorption spectrophotometry to analyze the concentration of six specific heavy metals: chromium (Cr), lead (Pb), cadmium (Cd), iron (Fe), antimony (Sb), and titanium (Ti).

According to the results shown in Table 2, Aneth seeds exhibit a high content of iron (Fe) at 0.5858 mg/L, followed by antimony (Sb) with a content of 0.0087 mg/L. Regarding the other detected heavy metals, it is noteworthy that they all fall within the allowable range set by FAO/WHO regulatory standards. Consequently, the studied plant is suitable for direct consumption, as an ingredient in food processing, or for repackaging if necessary.

### 2.2. Physical-Chemical Properties and Extraction Yield of Essential Oils

The data reported in Table 3 indicate that the essential oils (EOs) obtained from the seeds of *A. graveolens* gathered in the Errachidia region during full bloom were characterized by an aromatic scent, a yellowish hue, and a yield of 2.73 ± 0.12%.

Notably, this result was, on the one hand, significant compared to those from other origins: in Egypt 1.88% [17], in India 1.5% [9], in Iran (0.04–1.86%) [18], and also within the same country 1.92% [19]. On the other hand, it was lower than those reported elsewhere: in southern Morocco (3.5%) [20] and in China (6.7%) [21]. Based on the literature results and those obtained in this study, the variation in EO yield is attributed to various genetic, ecological, and environmental factors (plant age, climatic conditions, soil type, growth stage of the species, part used, harvest time, drying process, harvest period and environment, and cultural practices) as well as the extraction method used.

The plant under investigation yielded essential oils with a density of 0.9362 ± 0.0732 g/mL. Based on the AFNOR standard (2005), essential oils are advised to have a density ranging from 0.906 g/mL, which indicates lesser grade oils, to 0.990 g/mL, which is suggestive of oils of extremely high quality. The measurement indicates that the density of our essential oil is comfortably within the allowed range, indicating a quality that meets or exceeds the normal standards for essential oil density. This discovery suggests that the essential oil extracted from this plant possesses a praiseworthy quality that meets the standards set by the industry.

### 2.3. Chemical Composition of EO

The chemical profile (Supplementary Figure S1) revealed the presence of 29 chemical compounds for the total chemical composition of this essential oil. These results are summarized in Table 4.

The analysis of the chemical composition revealed that phenylpropanoids constituted the most abundant group of all identified compounds in the essential oil of *Anethum graveolens*, accounting for 67.93%, followed by monoterpenes divided between oxygenated monoterpenes (20.57%) and hydrogenated monoterpenes (11.36%). Hydrogenated sesquiterpenes were also identified but with a low percentage (0.14%).

**Figure 1:**
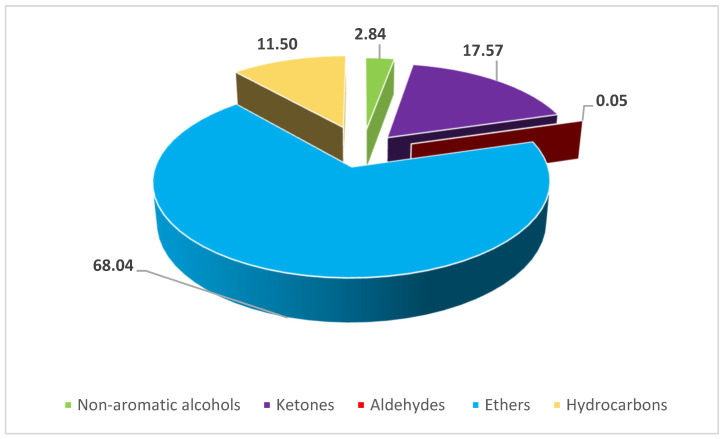
Distribution of the main chemical groups in the essential oil of *A. graveolens*.

Furthermore, the chemical composition of the *A. graveolens* essential oil was rich in ethers (68.04%), followed by ketones with a percentage of 17.57%. We also note the presence of hydrocarbons (11.5%) and alcohols (2.84%). However, aldehydes were nearly absent (0.05%) (Figure 1).

The essential oil extracted from the seeds of *A. graveolens* was mainly composed of (E)-anethole (38.13%), estragole (29.32%), fenchone (17.21%), and α-pinene (7.37%), while other compounds were present in smaller amounts, such as linalool (2.68%) and limonene (1.24%) (Table 4 and Supplementary Figure S2).

An analysis of the data revealed that the chemical composition of our *A. graveolens* essential oil differs from that reported in previous studies of other origins, with qualitative and quantitative differences in individual components being identified. Indeed, we observed that the studied plant exhibited an interesting chemical polymorphism.

In Morocco, few studies have focused on the chemical profile of dill essential oil. Znini et al. [20] identified 12 compounds representing 98% of the essential oil extracted from seeds from southern Morocco, with carvone (43.5%), dillapiole (26.7%), and limonene (15.4%) being the major compounds. Furthermore, chromatographic and spectrophotometric analyses conducted by El-Sayed et al. [17] on the essential oil extracted from Egyptian seeds revealed the following major compounds as the major constituents of dill essential oil: dillapiole (44.01%), D-limonene (19.47%), and carvotanacetone (13.03%). Meanwhile, in Tunisia, Snuossi et al. [22] reported carvone (27%), piperitone (25.7%), limonene (20.6%), dillapiole (8%), trans-dihydrocarvone (4.9%), and camphor (4.4%).

In Europe, French dill essential oil is mainly composed of limonene (48.05%), carvone (37.94%), cis-dihydrocarvone (3.5%), and trans-carvone (1.07%) [23]. Kostić et al. [24] reported the dominant presence of oxygenated monoterpenes (52.79%) and the total absence of phenylpropanoids in the composition of dill essential oil from Serbia. The latter contained a large quantity of carvone (42.47%), limonene (29.04%), and α-phellandrene (13.12%). Meanwhile, in Asia and Saudi Arabia, Aati et al. [25] found that the essential oil extracted from seeds, using the solid-phase microextraction method Headspace, was rich in monoterpenes (65.1%).

Moreover, its major compounds were anethole (33.3%), limonene (30.8%), carvone (17.7%), and trans-dihydrocarvone (12.2%). A study conducted reported that Iranian dill essential oil contains linalool (63.41%), δ-terpinene (4.27%), β-pinene (3.97%), p-cymene (3.35%), geranyl acetate (3.32%), octyl butyrate (3.3%), and α-pinene (3.23%) [26]. However, in the same country, Najafzadeh et al. [14] found that phellandrene (34.19%), carvone (23.67%), limonene (21.47%), α-terpineol (5.58%), and para-cymene (5.50%) were the main constituents of this species. In China, the major constituents of this studied essential oil are D-carvone (40.36%), D-limonene (19.31%), apiole (17.50%), α-pinene (6.43%), 9-octadecenoic acid (9.00%), and 9,12-octadecadienoic acid (2.44%) [21]. Similarly, in India, Kaur et al. [9] reported carvone (41.15%), limonene (23.11%), camphor (9.25%), and dihydrocarvone (3.75%) as the main compounds, while butyl acetate (2.65%), myrcene (2.365%), anethole (1.65%), α-pinene (1.06%), and aneth ether (1.02%) as the minor compounds in the essential oil of this species.

The various components reported by different studies also depend on the different growth stages of the plant, the cultivation area, and the specific part of the plant used.

### 2.4. Phytochemical Screening

Phytochemical tests were conducted on various extracts prepared from *A. graveolens* seeds using specific revealing reagents. The results of phytochemical screening are reported in Table 5.

This species was rich in primary metabolites, including polysaccharides, reducing sugars (glucose and fructose), proteins, and lipids at variable concentrations. Similarly, the presence of secondary metabolites, including flavonoids, tannins, mucilages, leucoanthocyanins, oses and holosides, sterols, and triterpenes, were confirmed. However, saponins and alkaloids were found to be absent.

Our results are consistent with those of El Mansouri et al. [27], who revealed the abundant presence of tannins and flavonoids, as well as the absence of saponins in dill seeds from Southern Morocco. Similarly, Kaur et al. [9] demonstrated the presence of phenolic compounds, flavonoids, tannins, and terpenoids.

### 2.5. Phenolic Compound Yields of Extracts

**Figure 2:**
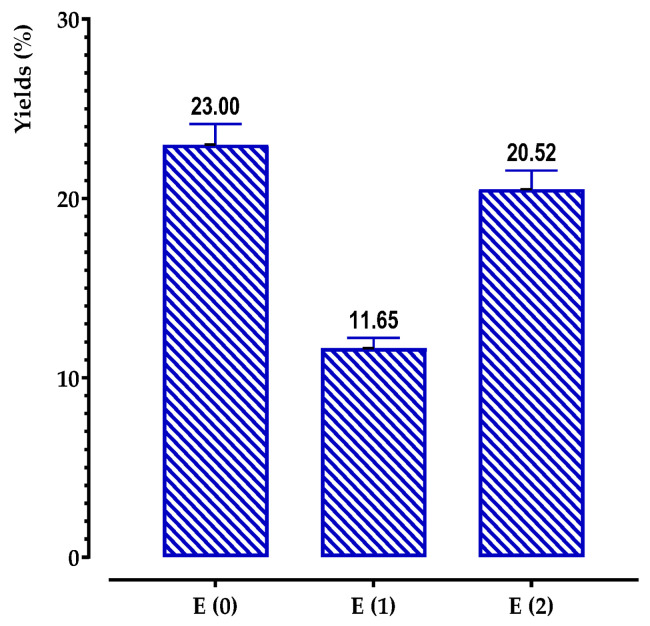
Yields of extracts obtained from *A. graveolens*.

We were able to determine the yield of several phenolic compounds found in the plant under investigation by extracting them. The extracts included ethanol, aqueous, and decocted extracts. The yield was calculated as a percentage relative to 30 g of the plant material that had been dried and powdered. The findings are depicted in Figure 2.

The results obtained showed that extraction yields depended both on the extraction solvent and the extraction method. Indeed, the average extraction yields of phenolic compounds were more or less variable and were higher in the decocted (23.00% ± 0.04) and aqueous extracts (20.52% ± 0.006) than in the ethanol extract (11.65% ± 0.003).

We observed a propensity for water to extract a greater number of chemicals compared to ethanol. This phenomenon may be attributed to the inherent property of water as a highly polar solvent, which has the ability to selectively extract a diverse array of molecules. This includes a substantial quantity of non-phenolic substances, such as sugars and proteins.

### 2.6. Content of Phenolic Compounds, Flavonoids, and Condensed Tannins

**Figure 3:**
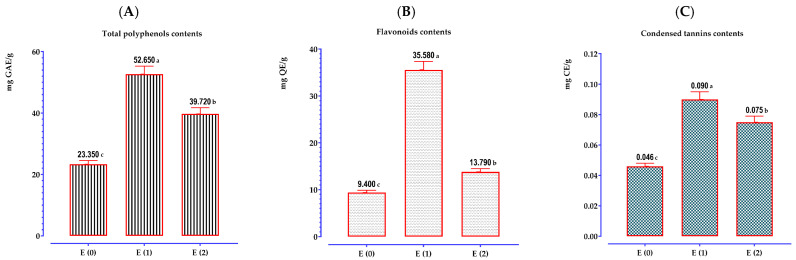
Contents of total polyphenols (**A**), flavonoids (**B**), and condensed tannins (**C**) in extracts from *A. graveolens* seeds. The values are means ± SD. Results with different superscripts are significantly different from each other (*p* < 0.05).

Figure 3 displays the outcomes acquired through the use of a UV–visible spectrophotometer pertaining to the concentrations of total phenolic components and flavonoids in extracts derived from *A. graveolens* seeds. Based on the observed data, it seemed that the amounts of these compounds exhibited significant variation between different extracts. The ethanolic extract recorded the highest quantities of phenolic compounds, flavonoids, and tannins at approximately 52.65 ± 0.22 mg GAE/g of extract, 35.58 ± 2.79 mg QE/g of extract, and 0.090 mg CE/g of extract, respectively, followed by the aqueous extract noting values of 39.72 ± 0.00 mg GAE/g of extract, 13.79 ± 0.63 mg QE/g of extract, and 0.075 ± 0.002 mg CE/g of extract. Meanwhile, the decoction procedure produced the lowest quantities of the tested molecules; notably, 23.35 ± 0.66 mg GAE/g of extract for phenolic compounds, 9.40 ± 0.51 mg QE/g of extract for flavonoids, and 0.046 ± 0.003 mg CE/g of extract for tannins. The results we obtained are significantly higher than those published by El Mansouri et al. [27]. Our research showed that the decoction had higher quantities of phenolic compounds and flavonoids but a somewhat reduced tannin content compared to a previous study. El Mansouri et al. [27], reported that the dill seed decoction had polyphenol and tannin concentrations of 6.16 mg GAE/g of extract and 0.21 mg CE/g of extract, respectively. This discrepancy underscores the fluctuation in phytochemical composition caused by factors such as geographic source, extraction procedure, and analytical methodologies.

### 2.7. Chromatographic Analysis by HPLC/UV-ESI-MS of the Extracts

The chromatographic profile, depicted below (Supplementary Figure S3), illustrates the peaks of compounds derived from the decoction of *A. graveolens* seeds, along with their retention times and relative abundances.

Using analytical and spectral data in negative and positive modes, we identified 38 compounds whose names and molecular formulas are presented in Table 6.

Analysis of the results obtained from the decoction revealed a structural diversity of molecules constituting the dill extract. Indeed, we detected the presence of several compounds, the most significant of which were two coumarins, umbelliferone (12.35%) and coumarin (4.21%), a 3-hydroxyflavone flavonoid (11.23%), a diterpene rosmanol (8.95%), a vitamin B7 biotin (8.36%), and a sesquiterpenoid emmotin H (4.91%).

ESI-MS, which stands for electrospray ionization-mass spectrometry, is a crucial technique for identifying the structure of compounds. It allows for the analysis of each component by fragmentation in both negative and positive ion modes.

The ESI-MS spectrum of umbelliferone showed that this molecule underwent ionization, resulting in the loss of a proton (H^−^), forming the [M-H]^−^ ion, observable at *m*/*z* = 161 and detected at 8.96 min in the mass spectrometer. Furthermore, the predominant fragments identified at *m*/*z* = 133 and 106 corresponded respectively to the separation of an acetate (CH_3_CO) group and a carbon monoxide (CO) group, thus characterizing the typical fragmentation of the coumarin structure. In a similar context, 3-hydroxyflavone (C_15_H_10_O_3_, M = 238) presented an [M-H]^−^ ion at *m*/*z* = 237, appearing at 9.91 min, accompanied by major fragments at *m*/*z* = 237, 135, and 101 that reflects the fragmentation of the flavonoid skeleton, including the release of a CO group and a C_5_H_4_O_2_ fragment. Moreover, rosmanol revealed an [M-H]^−^ ion at *m*/*z* = 345, observed at 10.15 min, with major fragments at *m*/*z* = 243 and 197, indicating the characteristic fragmentation of its polyphenolic structure.

Furthermore, biotin was detected by an [M+H]^+^ ion at *m*/*z* = 243, which appeared at 9.00 min, accompanied by fragments at *m*/*z* = 297, 228, 174, and 130 that testify to its specific fragmentation. The ion at *m*/*z* = 297 could result from the loss of a part of the biotin’s side structure, potentially involving the disappearance of a valerate group or a section of the imidazole. The fragment at *m*/*z* = 228 could result from the loss of a C_3_H_8_NS group, including a portion of the thiol group and the nitrogen. Finally, the ion at *m*/*z* = 174 could result from the loss of a C_4_H_6_NO_2_ group, likely associated with a section of the valeric acid or the imidazole.

Similarly, emmotin H (C_15_H_16_O_3_, M = 244) presented an [M-H]^−^ ion at *m*/*z* = 243, detected at 21.43 min, accompanied by fragments at *m*/*z* = 243, 228, 200, and 184, characteristic of the fragmentation-specific to its sesquiterpenoid structure. The ion at *m*/*z* = 243 corresponded to the parent [M-H]^−^ ion of the emmotin H molecule, revealing a loss of a proton during ionization, consistent with negative mass spectrometry processes. The fragment at *m*/*z* = 228 could result from the dissociation of a CH_2_ (methylene) group, a frequent observation in sesquiterpenoids. Furthermore, the fragment at *m*/*z* = 200 could indicate a more substantial loss in the carbon chain, possibly associated with the cleavage of an alkene group or another functional group. Finally, the ion at *m*/*z* = 184 could result from additional fragmentation of the sesquiterpenoid skeleton, likely involving the loss of a less complex structure.

Lastly, coumarin (C_9_H_6_O_2_, M = 146) showed an [M+H]^+^ ion at *m*/*z* = 147, identified at 9.24 min, accompanied by a principal fragment at *m*/*z* = 103, marking the distinctive fragmentation of its structure by the loss of a CO group.

Our results align with previous studies that have highlighted the richness of dill seeds in these molecules. Bota et al. [28] reported the presence of umbelliferone, kaempferol, and quercetin in hydromethanolic extracts of dill seeds from Romania. Similarly, Mashraqi et al. [29] observed the presence of phenolic acids and flavonoids such as chlorogenic acid, cinnamic acid, caffeic acid, rosmarinic acid, quercetin, and apigenin in dill from Saudi Arabia. In addition to these compounds, Erdogan et al. [30] revealed the presence of vanillic acid and the abundance of rosmarinic acid in dill seeds from Turkey. Furthermore, a bibliographic review by Meena et al. [31] noted the presence of vitamins B1 and B2 in dill seeds.

The pharmacological properties of coumarins are already well-documented in the literature, especially umbelliferone [32]. This coumarin is utilized as a sunscreen agent and possesses anti-inflammatory, antioxidant, antimicrobial, antihyperglycemic, molluscicidal, and antitumor activities [33].

Regarding flavonoids, apigenin, kaempferol, and quercetin, known for their various pharmacological effects, are used as dietary supplements due to their high antioxidant activity. Numerous preclinical studies have demonstrated their wide range of biological activities, including anti-inflammatory, antimicrobial, anticancer, and cardioprotective effects [34,35,36,37].

Clinical investigations have demonstrated that phenolic acids have beneficial effects on several significant pathological illnesses, including cancer, cardiovascular diseases, hepatotoxicity, neurotoxicity, and viral infections [38,39,40,41].

The chemical profile of the dill seed extract studied in our research explains, on the one hand, its numerous pharmacological properties proven by these cited studies, and on the other hand, confirms its use in traditional medicine. The chemical structures of the primary chemicals found in *A. graveolens* seeds are displayed in Supplementary Figure S4.

### 2.8. Antioxidant Activity of A. graveolens Essential Oil and Extracts

#### 2.8.1. Antioxidant Activity of Essential Oil Using DPPH

**Figure 4:**
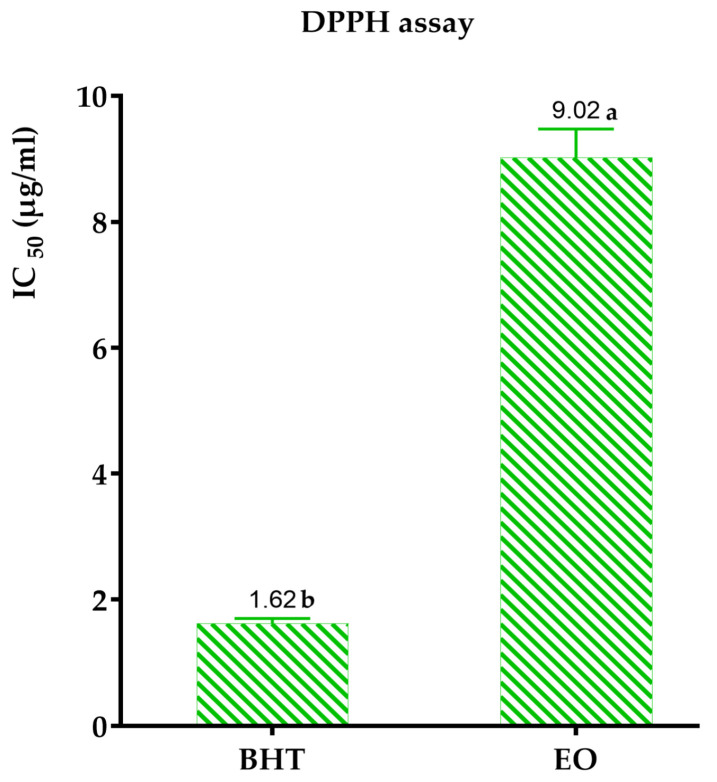
IC_50_ values of *A. graveolens* and the standard antioxidant BHT using the DPPH method. The results with different letters are significantly different from each other (*p* < 0.001).

The results obtained showed a concentration-dependent variation in the inhibition of DPPH* radicals by the *A. graveolens* essential oil. According to the IC_50_ values grouped in Figure 4, the standard antioxidant power of butylated hydroxytoluene (BHT) exceeded that of Dill. Indeed, the latter was considered less effective in reducing free radicals with an IC_50_ equal to 9.02 mg/mL.

This observation has been reported in several previous studies. For instance, Kaur et al. [9] identified through the DPPH method that Indian dill essential oil, mainly composed of carvone (41.15%), limonene (23.11%), camphor (9.25%), and dihydrocarvone (3.75%), exhibits low antioxidant activity (IC_50_ = 0.65 mg/mL) compared to the standard ascorbic acid (IC_50_ = 0.04 mg/mL). These authors attributed this antioxidant power to the presence of polar compounds. Similarly, Osanloo et al. [42] observed the low antioxidant activity of Iranian dill essential oil, whose major constituents are α-phellandrene (26.75%), p-cymene (24.81%), carvone (10.77%), dill ether (9.78), and cis-sabinol (3.61%), using the DPPH method. A study conducted in Serbia showed that dill essential oil, composed of limonene (45.24%) and carvone (45.90%), has higher antiradical activity than anise composed mainly of anethole (96.40%) [43]. Stanojević et al. [44] reported that dill essential oil from Serbia, primarily composed of carvone (85.9%), limonene (5.1%), and cis-dihydrocarvone (3.0%), inhibited 79.62% of DPPH radicals at a concentration of 29 mg/mL for 60 min.

The antioxidant activity of essential oils is determined by the collective interaction of their chemical components, which can operate either antagonistically or synergistically. Eos that include volatile phenolic compounds have potent antioxidant action as a result of their heightened reactivity with peroxyl radicals. Furthermore, in our work, the studied EO of dill was composed primarily of phenylpropanoids (67.93%). As a result, several previous studies have reported the antioxidant capacity of phenylpropanoids [45]. Additionally, the studied EO, composed of trans-anethole (38.13%), estragole (29.32%), and fenchone (17.21%), exhibited slight antioxidant activity. This low antioxidant activity has already been noted by Donati et al. [46]. These researchers have evaluated the antioxidant activity of trans-anethole and estragole using the DPPH and FRAP methods, and they have found their moderate antioxidant power. Moreover, another study conducted by Senatore et al. [47] has shown the low ability of anethole to scavenge DPPH free radicals and reduce ferric ions compared to the Trolox standard.

Moreover, Luís et al. [48] have tested the antioxidant activity of the EO of *I. Verum*, composed primarily of phenylpropanoids (92.2%), using the DPPH method. Crucially, these authors found that this EO, rich in trans-anethole (88.5%), has an IC_50_ (3.46%) that is nearly seventeen times higher than that of gallic acid (0.20%). Consequently, this suggests that the antioxidant potential of the *I. verum* EO is significantly lower compared to the standard gallic acid.

Furthermore, the antioxidant activity of the Indian dill essential oil was studied using DPPH (1,1-diphenyl-2-picrylhydrazyl) tests. Notably, the results have shown moderate antioxidant activity with inhibition percentages ranging from 26.1% to 43.62% for concentrations varying from 5 to 25 μL [49]. Additionally, Coêlho et al. [50] have revealed the antioxidant potential of estragole using DPPH and ABTS methods, but it remains lower than that of Trolox. Regarding fenchone, the work of El Omari et al. [51] has found, using the FRAP method, that it possesses a more pronounced reducing power than camphor and the essential oil of *Lavandula stoechas*. Similarly, Singh et al. [52] have highlighted the antioxidant effect of fenchone. Although these previous studies have demonstrated the antioxidant potential of trans-anethole, estragole, and fenchone, the moderate antioxidant activity of the studied dill EO is also attributed to the minor compounds, which may either strengthen or weaken its antioxidant activity through synergistic or antagonistic effects.

#### 2.8.2. Antioxidant Activity of Extracts

**Figure 5:**
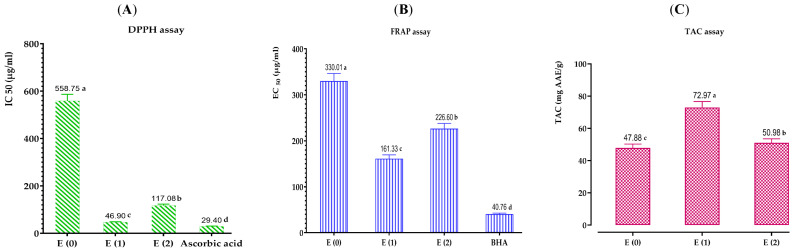
Antioxidant effects of extracts of *A. graveolens* by DPPH (**A**), and FRAP methods (**B**), and CAT (**C**). Mean values ± standard deviations of determinations performed in triplicate are reported; Means are significantly different (*p* < 0.001).

Based to the results presented in Figure 5, the antioxidant effects of extracts from *A. graveolens* are shown. The IC_50_ values noted for ascorbic acid and BHA, used as reference compounds by the DPPH and FRAP methods, were significantly lower than those of the extracts, indicating a high antioxidant activity of the standards.

Additionally, we can observe that the nature of the extraction solvent significantly affects the antioxidant activity. Notably, the ethanolic extract, which has shown high contents of polyphenols, flavonoids, and condensed tannins, has proven to be the most active, possessing a powerful antioxidant potential compared to the aqueous extract and decoction.

Moreover, all the tests have allowed us to highlight higher anti-radical ferric and molybdenum ion-reducing activities in the ethanolic extract than in the aqueous extract and decoction. Specifically, the IC_50_ value of this extract reaches 46.90 ± 0.73 μg/mL via the DPPH method, an EC_50_ equal to 161.33 ± 6.18 μg/mL via the FRAP method, and a total antioxidant capacity of 72.97 ± 0.71 mg EAA/g E.

Furthermore, for the aqueous extract, its ability to scavenge DPPH free radicals is superior to that of the decoction. This fact is confirmed by a lower IC_50_ of the aqueous extract (IC_50_ = 117.08 ± 0.16 μg/mL) compared to that of the decoction (IC_50_ = 558.75 ± 4.07 μg/mL). Similarly, for the FRAP and TAC methods, the aqueous extract indicated a higher capacity to reduce ferric and molybdenum ions than the decoction; the EC_50_ value was 226.60 ± 11.73 μg/mL, and the total antioxidant capacity reached 50.98 ± 0.2 mg EAA/g E. In contrast, the decoction had an EC_50_ of 330.01 ± 3.09 μg/mL and a total antioxidant capacity of 47.88 ± 1.73 mg EAA/g E.

Moreover, the difference in the antioxidant activity of the extracts is primarily related to the contents of polyphenols, flavonoids, and other aromatic compounds present. Based on our analysis of the phenolic compound assay results, we have discovered that the examined extracts contain substantial quantities of phenolic compounds. The ethanolic extract exhibits the highest levels of polyphenols, flavonoids, and condensed tannins, surpassing the aqueous extract and the commonly used decoction in traditional medicine.

Furthermore, with the help of the results from the chromatographic analyses conducted using HPLC/UV-ESI-MS for the decoction of dill seeds, we have identified a diverse chemical richness in its chemical profile, reflected by the presence of several chemical families such as coumarins, flavonoids, phenolic acids, and terpenoids. Its main compounds are umbelliferone (12.35%), 3-hydroxyflavone (11.23%), rosmanol (8.95%), vitamin B7 biotin (8.36%), emmotin H (4.91%), and coumarin (4.21%).

The antioxidant potential of *A. graveolens* seeds has been verified by several researchers in the literature. Specifically, Basavegowda et al. [53] have observed strong antioxidant activities of the methanolic extract of dill seeds from India. They have indicated, via the three radical scavenging methods of DPPH, hydroxyl, and nitric oxide, IC_50_ values of 19 ± 0.94, 28 ± 0.36, and 36 ± 0.64 μg/mL, respectively. Moreover, Al-oqail et al. [16] have shown, using the DPPH and hydrogen peroxide radical scavenging methods, that the methanolic extract of Saudi dill seeds has a considerable antioxidant capacity, with IC_50_ values of 225 and 126.3 μg/mL, respectively. Additionally, these researchers have found, using the FRAP method, that the maximum absorbance of this extract is 1.387 at a concentration of 1 mg/mL. Similarly, the study by El Mansouri et al. [27] has revealed the antioxidant power of the aqueous extract of Moroccan dill seeds using the DPPH, ABTS, and hydroxyl radical scavenging methods, as well as the reducing power using FRAP.

### 2.9. Correlation between Phenolic Compound Content and Antioxidant Activity of Extracts

The entirety of the previously obtained results highlights the relationship between the phenolic compounds and the antioxidant activity of *A. graveolens*. With this in mind, the linear correlation coefficients were calculated (Table 7).

The coefficients varied between 0.686 and 0.990. The total polyphenol contents of the extracts correlated strongly with the DPPH (R^2^ = 0.970), FRAP (R^2^ = 0.990), and CAT (R^2^ = 0.762) tests. Similarly, a positive correlation was observed between the flavonoids and the anti-radical activity, reducing activity, and total antioxidant capacity, with Pearson coefficients of R^2^ = 0.982, R^2^ = 0.957, and R^2^ = 0.964, respectively. The antioxidant activity recorded in our extracts is attributed to their richness in phenolic compounds. However, the condensed tannins are an exception. Although the ethanolic, aqueous, and decoction extracts did not possess high contents, there was a significant correlation in the case of FRAP (R^2^ = 0.969), DPPH (R^2^ = 0.938), and CAT (R^2^ = 0.686). This leads us to deduce that, while the quantity of condensed tannins is an important factor, it is not always sufficient, and there is another criterion related to condensed tannins that must be considered when interpreting antioxidant power, which is the quality criterion. Additionally, there was a positive linear correlation between the three evaluated antioxidant activities, either by the DPPH test and the CAT test for R^2^ = 0.896, between the DPPH test and the FRAP test for R^2^ = 0.995, or between the CAT test and the FRAP test for R^2^ = 0.845. This observation likely indicates that in the three extracts, the polyphenols, flavonoids, and condensed tannins are the major compounds involved in the anti-radical activity, reducing activity, and total antioxidant capacity.

### 2.10. Antimicrobial Activity

Table 8 and Table 9 present the minimum inhibitory concentrations (MICs), minimum fungicidal concentrations (MFCs), and minimum bactericidal concentrations (MBCs) expressed in mg/mL for the extracts and in μL/mL for the essential oil of *Anethum graveolens* against the tested microorganisms.

According to the obtained results, the dill extracts and essential oil tested against the four fungi have revealed a promising antifungal activity, except for the decoction, which is inactive. For the ethanolic and aqueous extracts, the MICs and MFCs ranged between 0.78 and 50 mg/mL. Notably, the fungus *Aspergillus niger* was the most sensitive, with MICs and MFCs of 0.78 mg/mL. The highest MICs and MFCs were 50 mg/mL, which were obtained against *C. albicans*. Regarding *C. parapsilosis*, the ethanolic and aqueous extracts exerted fungistatic and fungicidal effects at the same concentration of 12.5 mg/mL. Furthermore, we also observed that the ethanolic extract exhibited a higher antifungal power compared to the other tested extracts. This high activity can be directly attributed to its elevated contents of polyphenols and flavonoids.

Regarding the essential oil, the MICs and MFCs ranged between 3.125 and 6.25 μL/mL. Notably, the fungi *A. niger* and *C. dubliniensis* were the most sensitive, with MICs and MFCs of 3.13 μL/mL. The highest MICs and MFCs of 50 μL/mL were observed in *Candida tropicalis* and *Candida parapsilosis*.

Regarding the antibacterial activity, the dill extracts and essential oil appeared to be sensitive to various bacterial strains to varying degrees. Specifically, the aqueous and decoction extracts presented bacteriostatic and bactericidal actions at a concentration of 50 mg/mL on *K. pneumoniae* and *S. aureus*. Moreover, the ethanolic extract exhibited a similar action on all the tested bacterial strains except for the *Enterobacter cloacae* strain, where it was inactive. Additionally, the three tested extracts did not exert any power on wild-type *E. coli* and *S. epidermidis*. In contrast, the essential oil inhibited and destroyed the *E. cloacae*, *K. pneumoniae*, and wild-type *E. coli* strains at a concentration of 25 μL/mL. Meanwhile, *S. aureus* and *S. epidermidis* appeared to be less sensitive, with higher MBCs of 50 μL/mL.

These results corroborate those found by Kaur et al. [54]. These authors have highlighted the antibacterial potential of the aqueous extract of Indian dill seeds against *E. coli* and *S. aureus*, noting MICs of 40 and 20 mg/mL, respectively. Additionally, another study has revealed the antimicrobial potential of the essential oils extracted from different parts: the leaves, stems, flowers, and seeds of *A. graveolens* from Saudi Arabia. Furthermore, this study indicated that the essential oil of the seeds, composed of dillapiole (33.3%), limonene (30.8%), carvone (17.7%), and trans-dihydrocarvone (12.2%), exhibited a higher antimicrobial power than the essential oils from the other parts against *S. aureus*, *C. albicans*, and *C. parapsilosis* [25]. Basavegowda et al. [53] have reported the antibacterial effect of the methanolic extract of dill seeds from India against *E. coli*, *K. pneumoniae*, and *S. aureus*, with MICs of 1250, 833, and 125 μg/mL, respectively. Generally, the observed antimicrobial power of the essential oil is closely linked to its chemical composition. Moreover, Mota et al. [55] have attributed the distinguished antibacterial activity of the essential oil of *Foeniculum vulgare* seeds to the synergistic effects of the major compounds present in an equivalent percentage, namely, (E)-anethole (37.2%), estragole (31.1%), and fenchone (28.5%).

Accordingly, in our study, the analyzed essential oil contained three main compounds in an almost equivalent percentage, namely, (E)-anethole (38.13%), estragole (29.32%), and fenchone (17.21%). Consequently, we can attribute the antimicrobial effect to the synergistic effects among the principal compounds present in this complex mixture. Furthermore, the observed antimicrobial potential can also be related to the richness of the studied essential oil in ethers (68.04%) and the presence of ketones (17.57%).

### 2.11. Prediction of PASS Activity, ADME, Toxicity (ProTox II), and Efficacy for Potentially Active Compounds Isolated from the Essential Oil and Aqueous Extract of A. graveolens Seeds

Examining the physicochemical features of the candidate compounds is crucial for developing therapeutic agents and confirming the effectiveness of *A. graveolens* as a nutraceutical preservative agent, in addition to its biological qualities. Therefore, an analysis was conducted on the chemicals found in the essential oil and aqueous extract of *A. graveolens* seeds to determine their pharmacokinetic and physicochemical characteristics, as well as their similarity to medications.

The main compounds of the EO (E-anethole, estragole, fenchone, and α-pinene) and the aqueous extract (umbelliferone, 3-Hydroxyflavone, rosmanol, biotin, emmotin H, coumarin, trans-caftaric acid, pimelic acid, methyl rosmarinate, homovanillic acid, 1-caffeoyl-beta-D-glucose, and kaempferol) of *A. graveolens* were selected for PASS and ADMET (absorption, distribution, metabolism, excretion, and toxicity) prediction studies.

The SMILES formats of these molecules were generated utilizing the ChemBio-Draw web tool [56], and subsequently simulated with the PASS web prediction tool [57] and the SwissADME and pkCSM [58] web tools. The outcomes of the PASS and ADMET prediction studies are presented in Table 10.

The efficiency of antioxidant and antibacterial properties of the major components extracted from the EO and aqueous extract of *A. graveolens* seeds was evaluated using PASS predictions.

Based on our projections, all the important chemicals had substantial “Pa” values for different activities, including antioxidant (0.150–0.856), antifungal (0.267–0.717), and antibacterial potential (0.216–0.587) (Table 10). Moreover, these compounds had highly favorable antioxidant characteristics, a potent antifungal impact, and commendable antibacterial activities.

The pkCSM and SwissADME websites are useful for analyzing the pharmacokinetic properties and the similarity of certain compounds to drugs. The lipophilicity indices of all the selected compounds indicate that they are quite water-soluble (Table 10).

The chosen substances present favorable values of skin permeability (log Kp) and high Caco-2 permeability values. Consequently, most of the studied compounds show excellent intestinal absorption (HIA > 30%), except for trans-caftaric acid.

The P-glycoprotein, or P-gp, plays a crucial role in the distribution and absorption of drugs. None of the chemical compounds of the studied essential oil act as inhibitors of P-gp I and P-gp II, and the main constituents of the essential oil are also not P-gp substrates. In contrast, for the studied compounds in the aqueous extract, we observed that compounds such as 3-hydroxyflavone, rosmanol, methyl rosmarinate, homovanillic acid, 1-caffeoyl-beta-D-glucose, and kaempferol can be P-gp substrates. Additionally, only rosmanol could act as an inhibitor of P-gp I.

The majority of the isolated compounds studied, eight out of a total of sixteen, obtained an SNC score greater than -3.0, suggesting that they easily cross the blood-brain barrier (BBB). However, certain exceptions were noted, particularly for biotin, emmotin H, coumarin, trans-caftaric acid, pimelic acid, methyl rosmarinate, homovanillic acid, and 1-caffeoyl-beta-D-glucose, which did not reach this threshold. Among the compounds with weak penetration into the central nervous system (log BB > 0.3), we found the compounds from the studied essential oils as well as 3-hydroxyflavone from the aqueous extract. Their volume of distribution in tissues (logVDss) ranged from -1.533 L/kg to 1.274 L/kg.

The main constituents of the essential oil and the aqueous extract examined are unlikely to have negative effects when taken orally due to drug interactions; however, cytochrome P450 (CYP) enzymes and molecular interactions play a crucial role in the elimination of drugs. The retinal organic cation transporters 2 (OCT2) and the total clearance of hepatic and renal substrates (CLTOT) were represented in log mL/min/kg to predict the excretion pathway. All the studied phytochemical compounds presented good overall clearance values and were excreted according to the data.

In order to evaluate the potentially harmful effects of the essential oil and aqueous extract of *A. graveolens* seeds, various aspects such as AMES, hERG channel inhibition, skin sensitization, immunotoxicity, hepatotoxicity, carcinogenicity, mutagenicity, and cytotoxicity were examined. The analysis focused on the main phytochemical constituents, as shown in Table 11. The data indicated the absence of significant toxic effects, except for certain molecules. Among these compounds, Biotin may have minimal hepatic effects; (E)-anethole, estragole, umbelliferone, 3-hydroxyflavone, and coumarin may have minimal carcinogenic effects. However, rosmanol, trans-caftaric acid, methyl rosmarinate, and 1-Caffeoyl-beta-D-glucose may present immunotoxicity effects. Finally, umbelliferone, also known as 7-hydroxycoumarin, is a natural organic compound widely distributed in the coumarin family with an LD_50_ of 10,000 mg/kg, classified in the predicted toxicity class 6. This compound is one of the main constituents of the aqueous extract of the studied *A. graveolens* seeds. These findings indicate that as long as the suggested doses are followed, the essential oil and aqueous extracts obtained from *A. graveolens* seeds from southern Morocco can be considered safe for oral consumption as nutraceuticals and/or as natural substitutes for synthetic preservatives. However, further research, including rigorous clinical trials, would be necessary to conclusively verify that there are no long-term risks to consumers.

### 2.12. Molecular Docking

Considering the much-increased in vitro bioactivities shown in the EO and aqueous extract of *A. graveolens* in this work, the compounds discovered using GC/MS and HPLC/UV-ESI-MS were chosen for in silico molecular docking experiments.

The antioxidant, antifungal, and antibacterial activities of the compounds, as well as their potential mechanism of action, were deduced through molecular docking experiments. These deductions were based on the molecular interactions at the atomic level between the compounds and their respective target proteins (1JZQ, 1KZN, 2CAG, 2VEG, 2ZDQ, 3SRW, 3UDI, 4URN, 2CDU, 1OG5, and 3NRZ). The molecular docking study was mostly concentrated on assessing factors such as Van der Waals (VDW) interactions, hydrogen bonds, binding free energy, and carbon–hydrogen (C–H) bonds. The stability of the ligands (chosen molecules) and the docked receptor is determined by the C–H bonds and Pi-sigma interactions, whereas the binding interactions are influenced by hydrogen bonds and Van der Waals (VDW) interactions. Table 12 displays the docking energy binding scores for the binding sites of the target proteins involved in antibacterial activities (1JZQ, 1KZN, 2CAG, 2VEG, 2ZDQ, 3SRW, 3UDI, and 4URN), as well as antioxidant activities (2CDU, 1OG5, and 3NRZ). This study analyzed the Van der Waals interactions, hydrogen bonds, and C–H bonds involving the amino acids found in the binding sites of the selected proteins.

The results of our study reveal that among the tested compounds, 3-hydroxyflavone, emmotin H, trans-caftaric acid, methyl rosmarinate, 1-caffeoyl-beta-D-glucose, and kaempferol presented the greatest binding energies with the exploratory proteins. These compounds demonstrated significant interactions with different target proteins involved in antimicrobial and antioxidant activities. In particular, 3-Hydroxyflavone exhibited a marked affinity with the 2CAG protein (−9.4 Kcal/mol) for antimicrobial activity. Similarly, Emmotin H manifested substantial binding energy with the 2ZDQ protein in this same context (−10 Kcal/mol). Moreover, Caftaric <trans> acid also displayed a strong affinity with the 2ZDQ protein (−9.1 Kcal/mol) for antimicrobial activity.

On the other hand, Methyl rosmarinate revealed significant binding energies with the 2CAG (−10.2) and 2ZDQ (−9.2 Kcal/mol) proteins for antimicrobial activity, as well as with the 3NRZ (−9.8 Kcal/mol) protein for antioxidant activity. Similarly, 1-Caffeoyl-beta-D-glucose presented high affinity with the 2CAG (−9.5 Kcal/mol) and 2ZDQ (−9.8 Kcal/mol) proteins for antimicrobial activity, as well as with the 3NRZ (−10 Kcal/mol) protein for antioxidant activity. Finally, Kaempferol also demonstrated substantial binding energy with the 2CAG protein (−10 Kcal/mol) for antimicrobial activity. These results highlight the promising potential of these bioactive compounds as antimicrobial and antioxidant agents, thus paving the way for future research in the fields of pharmaceutical chemistry and natural products.

**Figure 6:**
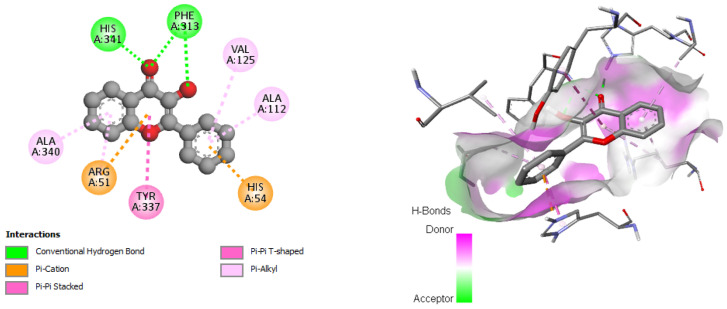
2D and 3D Interactions of 3-Hydroxyflavone with the 2CAG Target Protein.

The study of interaction types using Discovery Studio revealed that the interactions of 3-Hydroxyflavone with Catalase were associated with hydrogen bonds, Pi-alkyl, Pi-cation, and Pi–Pi-stacked with each of these residues (HIS341, PHE313, VAL125, ALA112, ALA340, ARG51, HIS54, and TYR337, respectively) (Figure 6).

**Figure 7:**
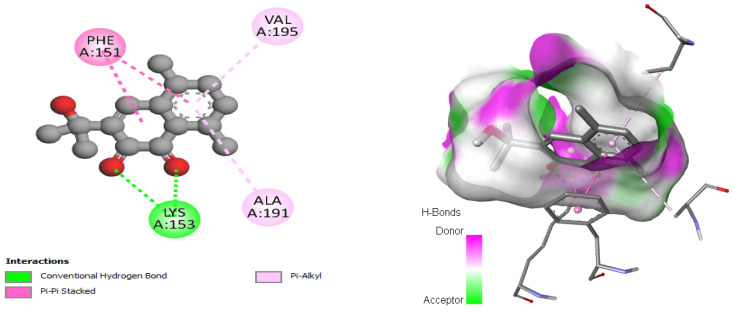
2D and 3D interaction of Emmotin H with D-Alanin Ligase protein.

This interaction study revealed that in the case of the D-alanine ligase protein, Emmotin H developed hydrogen bonds, Pi–Pi stacking, and Pi–alkyl interactions with each of the residues (LYS153, PHE151, VAL195, and ALA191, respectively) at their binding site on the D-Alanine Ligase (Figure 7).

**Figure 8:**
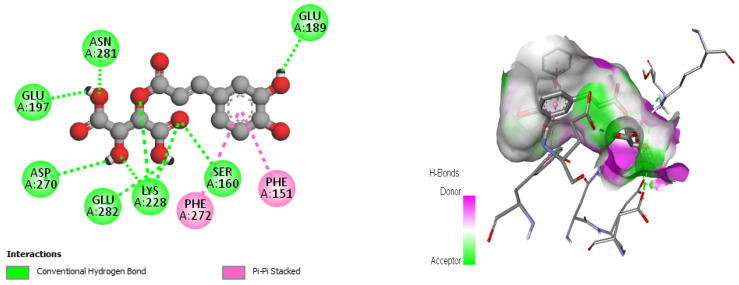
2D and 3D Interactions of trans-caftaric Acid with the 2ZDQ Protein.

Furthermore, in the case of trans-caftaric acid, hydrogen bonds and Pi–Pi stacking interactions were observed with each of the residues (GLU189, ASN281, GLU197, ASP270, GLU282, LYS228, SER160, PHE272, and PHE151) (Figure 8).

**Figure 9:**
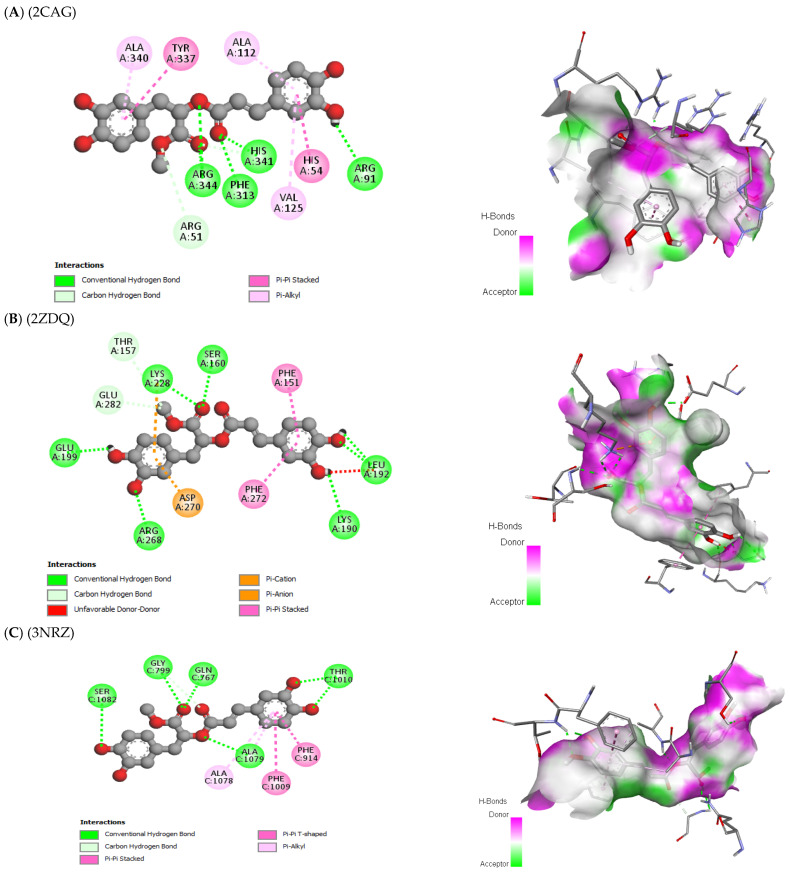
2D and 3D interaction of methyl rosmarinate with target proteins: (**A**) 2CAG, (**B**) 2ZDQ, and (**C**) 3NRZ.

For each of these catalytic residues (ARG344, PHE313, HIS341, ARG91, ARG51, TYR337, HIS54, VAL125, ALA340, and ALA112), the interaction of Methyl rosmarinate with them involved hydrogen bonds, carbon–hydrogen bonds, Pi–Pi stacked interactions, and Pi–alkyl interactions (Figure 9). On the other hand, the interactions of this molecule with D-Alanine ligase were associated with hydrogen bonds, carbon–hydrogen bonds, Pi–cation, Pi–anion, and Pi–Pi stacked interactions with each of the involved residues (GLU199, ARG268, LYS190, LEU192, LYS228, SER160, THR157, GLU282, ASP270, PHE151, and PHE272) (Figure 9). Finally, the interactions of Methyl rosmarinate with 3NRZ were characterized by hydrogen bonds, carbon–hydrogen bonds, Pi–Pi stacked, Pi–Pi-shaped, and Pi–alkyl interactions with each of the concerned proteins (SER1082, GLY799, GLN767, ALA1079, THR1010) (Figure 9).

**Figure 10:**
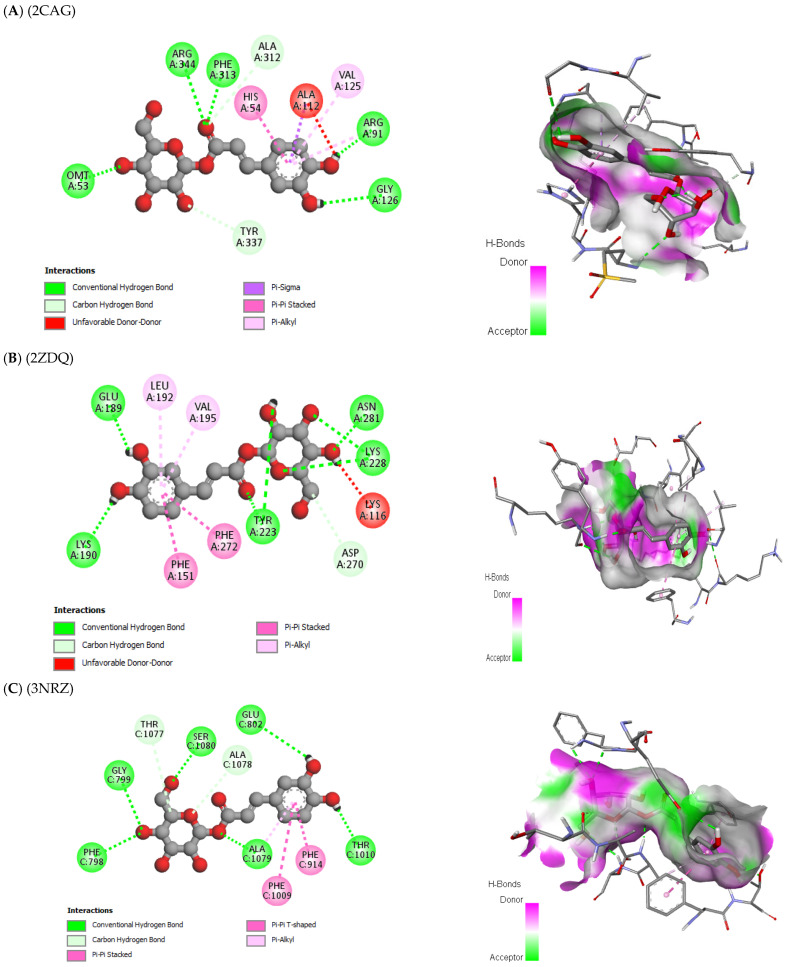
2D and 3D interaction of 1-Caffeoyl-beta-D-glucose with target proteins: (**A**) 2CAG, (**B**) 2ZDQ, and (**C**) 3NRZ.

For each protein (OMT53, ARG344, PHE312, GLY126, ALA312, TYR337, ALA112, HIS54, and VAL125), the interaction of 1-Caffeoyl-beta-D-glucose with catalase was associated with hydrogen bonds, carbon–hydrogen bonds, Pi–sigma interactions, Pi–Pi stacked interactions, and Pi–alkyl interactions (Figure 10). Furthermore, the interactions of 1-Caffeoyl-beta-D-glucose with D-Alanine ligase were characterized by hydrogen bonds, carbon–hydrogen bonds, Pi–Pi stacked interactions, and Pi–alkyl interactions with each of the involved proteins (LYS190, GLU189, ASN281, LYS228, TYR223, ASP270, LYS116, PHE151, PHE272, LEU192, and VAL195) (Figure 10). Finally, the interactions of 1-Caffeoyl-beta-D-glucose with bovine xanthine oxidase were associated with hydrogen bonds, carbon–hydrogen bonds, Pi–Pi stacked, Pi–Pi-shaped, and Pi–alkyl interactions with each of the concerned proteins (PHE798, GLY799, SER1080, GLU802, THR1010, ALA1079, THR1077, ALA1078, PHE914, and PHE1009) (Figure 10).

**Figure 11:**
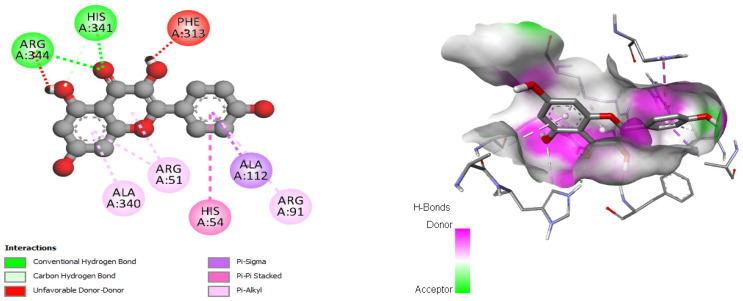
Interaction of 2CAG with kaempferol.

In conclusion, the interaction analysis has highlighted that the interactions of Kaempferol with the catalase protein were characterized by hydrogen bonds, carbon–hydrogen bonds, Pi–sigma interactions, Pi–Pi stacked interactions, and Pi–alkyl interactions with each of the protein residues (ARG344, HIS341, PHE313, ALA112, HIS54, ALA340, ARG51, and ARG91, respectively) (Figure 11).

### 2.13. Molecular Dynamics Simulation

It is worth noting that during an interaction with a drug molecule, a protein target can undergo significant conformational changes. Molecular dynamics simulation (MDS) serves as an effective tool for understanding internal motions, conformational alterations, and the stability of protein–ligand complexes. Analyzing MDS trajectories for the ten complexes, with two complexes in the case of 3NRZ, four complexes in the case of 2CAG, and four complexes in the case of 2ZDQ, allows for the computation of parameters such as root-mean-square deviation (RMSD), root-mean-square fluctuation (RMSF), radius of gyration (Rg), number of hydrogen bonds, and binding free energy. These analyses provide insights into the structural stability, binding modes, and binding strengths of the complexes.

#### 2.13.1. Structural Dynamics of 3NRZ

**Figure 12:**
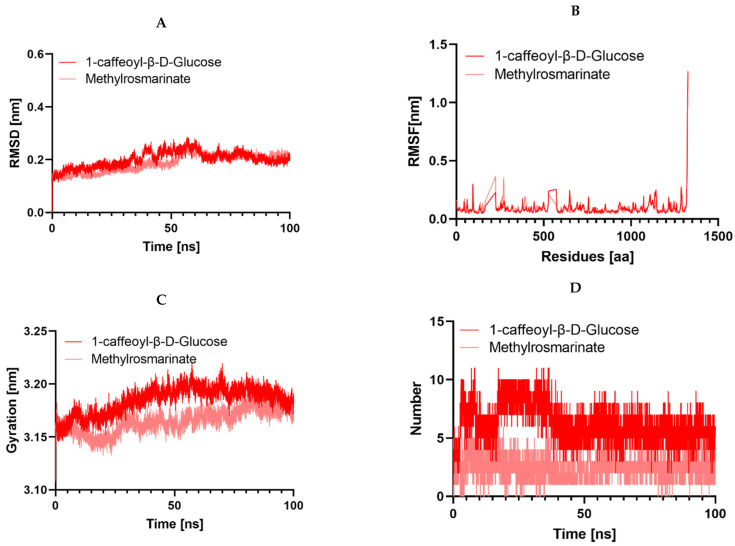
Structural dynamics of the 3NRZ protein: (**A**) RMSD and (**B**) RMSF. (**C**) Total number of intramolecular hydrogen bonds. (**D**) Radius of gyration.

Our study employed molecular dynamics simulations to investigate the complex structural dynamics of protein 3NRZ when interacting with methyl-rosmarite and 1-Caffeoyl-beta-D-glucose ligands. We aimed to understand the subtle effects of ligand binding on the stability of the protein’s conformation. We focused on various measures to comprehend how the protein and ligands interact dynamically. Upon examining the RMSD graph displayed in Figure 12, it can be observed that all the protein–ligand entities had a reduced variation in their spectrum. This indicates that there was a minimal disturbance in their conformational dynamics over the whole simulation. The RMSF analysis investigated ligand binding effects on protein dynamics over 100 ns, revealing decreased flexibility and lower potential conformational changes within protein–ligand complexes. This suggests that all complexes have a minimal impact on receptor flexibility, as depicted in Figure 12. Furthermore, examination of the Radius of Gyration (RG) profiles demonstrated consistent trends in both complexes, suggesting the maintenance of a stable conformation. Notably, hydrogen bonding analysis unveiled the formation of multiple hydrogen bonds in both complexes, underscoring their pivotal role in maintaining stability, even amidst ligand dissociation.

#### 2.13.2. Structural Dynamics of 2CAG

**Figure 13:**
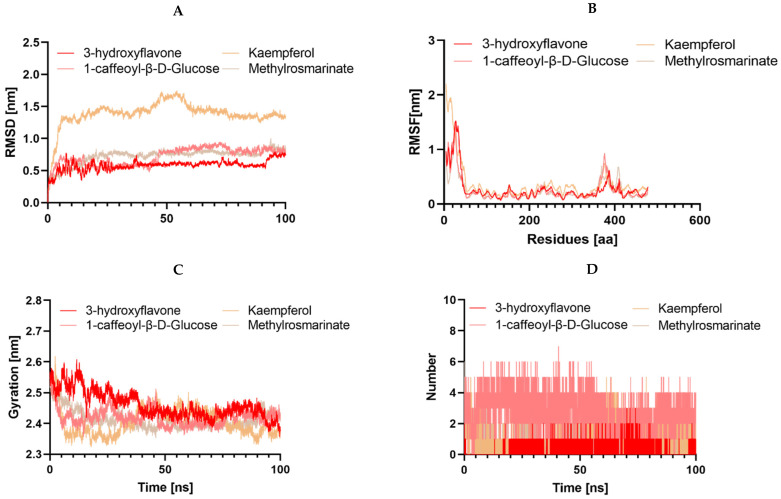
Structural dynamics of the 2CAG protein: (**A**) RMSD and (**B**) RMSF. (**C**) Total number of intramolecular hydrogen bonds. (**D**) Radius of gyration.

In this study, we delved into the structural dynamics of 2CAG through molecular dynamics simulations, focusing on their interaction with various ligands (Figure 13). Our analysis covered four different ligands (3-hydroxyflavone, Methylrosmarinate, 1-caffeoyl-β-D-Glucose, and Kaempferol), shedding light on their effects on protein conformation and dynamics. Notably, we observed strikingly similar RMSD profiles across the protein-ligand complexes, indicating minimal conformational changes upon ligand binding, except for Kaempferol, which displayed a notably higher RMSD. This suggests that while 3-hydroxyflavone, Methylrosmarinate, and 1-caffeoyl-β-D-Glucose induce negligible structural rearrangements in the protein backbone, Kaempferol triggers more pronounced changes, possibly reflecting a distinct binding mode or affinity. Further analysis of RMSF patterns revealed consistent dynamics among complexes 2CAG/3-hydroxyflavone, 2CAG/Methylrosmarinate, 2CAG/1-caffeoyl-β-D-Glucose, while the 2CAG/Kaempferol complex exhibited heightened flexibility, implying differential impacts of the ligands on protein flexibility. Examination of protein compactness using the radius of gyration (Rg) showed that 3-hydroxyflavone, Methylrosmarinate, and 1-caffeoyl-β-D-Glucose led to higher protein compactness compared to Kaempferol, correlating with the RMSD findings and suggesting tighter binding of the protein structure. Additionally, simulations unveiled the formation of hydrogen bonds between 3-hydroxyflavone, Methylrosmarinate, and 1-caffeoyl-β-D-Glucosethe target proteins over a 100 ns period, indicative of significant molecular interactions conducive to stable binding. However, Kaempferol formed fewer hydrogen bonds, implying weaker or less favorable interactions with the protein.

#### 2.13.3. Structural Dynamics of 2ZDQ

**Figure 14:**
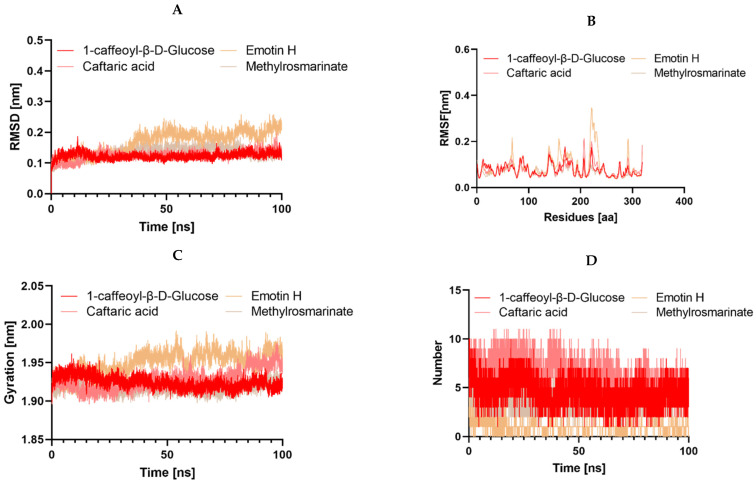
Structural dynamics of the 2ZDQ protein: (**A**) RMSD and (**B**) RMSF. (**C**) Total number of intramolecular hydrogen bonds. (**D**) Radius of gyration.

In this case, we explored the structural dynamics of 2ZDQ via molecular dynamics simulations, with a specific focus on how it interacts with four distinct ligands: emmotin H, trans-caftaric acid, methyl rosmarinate, and 1-caffeoyl-β-D-glucose. This investigation sheds light on their effects on protein conformation and dynamics. Notably, we observed strikingly similar RMSD profiles across the protein–ligand complexes, indicating minimal conformational changes upon ligand binding, except for emmotin H, which displayed a notably higher RMSD. This suggests that while trans-caftaric acid, methyl rosmarinate, and 1-caffeoyl-β-D-glucose induce negligible structural rearrangements in the protein backbone, emmotin H triggers more pronounced changes, possibly reflecting a distinct binding mode or affinity. Further analysis of RMSF patterns showed that complexes formed with trans-caftaric acid, methyl rosmarinate, or 1-caffeoyl-β-D-glucose had similar dynamics. However, the emmotin H-2ZDQ complex showed increased flexibility, suggesting that the ligands had varied effects on the flexibility of the protein. Next, we investigated the changes in the protein’s structural compactness resulting from its interaction with these various compounds. To achieve this, we computed the radius of gyration (Rg) over time (Figure 14). Consistent with the results of the RMSD analysis of the protein backbone, the examination of protein compactness using the Rg showed that trans-caftaric acid, Methyl rosmarinate, and 1-caffeoyl-β-D-Glucose led to higher protein compactness compared to emmotin H. Furthermore, simulations revealed the establishment of hydrogen bonds over 100 ns between trans-caftaric acid, methyl rosmarinate, or 1-caffeoyl-β-D-glucose and the target protein, indicating substantial molecular interactions favorable for persistent binding. However, emmotin H created fewer hydrogen bonds, which suggests that its interactions with the protein were weaker or less advantageous.

To design therapeutic agents and support the efficacy of the chemical composition of *A. graveolens* as a nutraceutical preservative, it is essential to analyze in detail the physicochemical characteristics of potential compounds, as well as the pharmacokinetic and physicochemical properties of the substances identified in the essential oil (EO) and aqueous extract (E_0_) of *A. graveolens* seeds, comparing them to drugs. The predictive PASS and ADMET evaluations of the main constituents of the EO and aqueous extract (AE) of *A. graveolens* revealed significant values for various activities, such as antioxidant, antifungal, and antibacterial properties.

Previous research, particularly that of Najaran et al. [59], Noumi et al. [23], as well as the work of Madhuri [60], confirm the antioxidant and antimicrobial capabilities of the compounds contained in *A. graveolens*, thus suggesting their potential as therapeutic agents for various ailments. These findings highlight the exceptional qualities of the compounds identified in the EO and aqueous extract (E_0_) of *A. graveolens*, which exhibit notable antioxidant, antifungal, and antibacterial activities.

The main phenolic, flavonoid, and terpenoid compounds extracted from *A. graveolens* seeds offer a wide range of pharmacological and biological applications. These compounds demonstrate promising antimicrobial, antioxidant, and anticancer properties in preclinical studies. These molecules, such as methyl rosmarinate, trans-caftaric acid, 1-caffeoyl-beta-D-glucose, kaempferol, and 3-hydroxyflavone, are currently being investigated for their biological activity. Methyl rosmarinate stands out for its antioxidative properties and antifungal activities.

It exhibits inhibitory activities against tyrosinase, α-glucosidase, and matrix metalloproteinase-1 (MMP-1) [61,62,63,64]. Trans-caffeic acid shows promise in antioxidant, anti-inflammatory, antimutagenic, anticarcinogenic, hepatoprotective, antidiabetic, antihypertensive, anti-obesity, and metabolic syndrome, as well as neuroprotective effects [65]. 1-Caffeoyl-beta-D-glucose, kaempferol, and 3-hydroxyflavone display a variety of pharmacological activities, suggesting a significant role in several therapeutic areas [66,67].

The results of our molecular docking reveal significant interactions between several bioactive compounds and various target proteins involved in antimicrobial and antioxidant activities. Among these compounds, 3-hydroxyflavone, emmotin H, trans-caftaric acid, methyl rosmarinate, 1-caffeoyl-beta-D-glucose, and kaempferol exhibited the highest binding energies with the explored proteins. These compounds have demonstrated a strong affinity with specific proteins, such as 2CAG, 2ZDQ, and 3NRZ, indicating their potential as antimicrobial and antioxidant agents. Furthermore, the study of molecular interactions using 3-hydroxyflavone revealed hydrogen bonds, Pi–alkyl bonds, Pi–cation interactions, and Pi–Pi-shaped interactions with specific catalase residues. Similarly, emmotin H formed hydrogen bonds, Pi–Pi stacking, and Pi–alkyl interactions with D-Alanine ligase residues, while trans-caftaric acid formed hydrogen bonds and Pi–Pi stacking with the latter’s residues.

These specific interactions highlight the diversity of the action modes of the bioactive compounds on the target proteins. Furthermore, the analysis of catalytic residues involved in interactions with Methyl rosmarinate revealed the presence of hydrogen bonds, carbon–hydrogen bonds, Pi–Pi stacked interactions, and Pi–alkyl interactions. Additionally, interactions of this molecule with D-Alanine ligase were characterized by hydrogen bonds, carbon–hydrogen bonds, Pi–cation interactions, Pi–anion interactions, and Pi–Pi stacked interactions. Finally, interactions with 3NRZ showed hydrogen bonds, carbon-hydrogen bonds, Pi–Pi-stacked interactions, Pi–Pi-shaped interactions, and Pi–alkyl interactions. Regarding 1-Caffeoyl-beta-D-glucose, its interactions with catalase, D-Alanine ligase, and bovine xanthine oxidase were associated with different bonds, such as hydrogen bonds, carbon–hydrogen bonds, Pi–sigma interactions, Pi–Pi stacked interactions, and Pi–alkyl interactions.

Furthermore, the analysis of structural dynamics using molecular dynamics simulations provided insights into conformational changes and the stability of protein–ligand complexes [68]. These simulations revealed similar dynamic behaviors among certain complexes, as well as significant differences, suggesting distinct interaction modes and variable effects on protein flexibility and stability.

These results underscore the diversity of molecular interactions between bioactive compounds and target proteins, thus paving the way for future research in the fields of pharmaceutical chemistry and natural products.

Ultimately, despite their promising therapeutic potential, these compounds still require FDA approval for medical use, highlighting the need for further research, particularly clinical trials, to assess their efficacy and safety in patients in combating oxidative stress. These molecules are being explored for their antioxidant potential and could act as scavengers of free radicals, thereby protecting cells against oxidative stress-induced damage by modulating the expression of specific cytokines and enzymes such as SOD, dehydratase, CAT, GST, GSH, GPx, and GRd.

The antioxidant activity of *A. graveolens* essential oil, as determined by the DPPH technique and quantified by an IC_50_ value of 9.02 mg/mL, might potentially be elucidated by the findings of molecular docking investigations. Hence, compounds like trans-anethole and fenchone, which have exhibited substantial interactions with specific proteins associated with antioxidant processes, such as superoxide dismutases or catalases, may be accountable for the reported antiradical activity in laboratory settings. These compounds have electrical and structural features that are advantageous for neutralizing free radicals. This is mainly due to their phenolic or terpenic groups, which may efficiently remove reactive oxygen species.

The antifungal and antibacterial activities of the extracts and essential oils were observed to have minimum inhibitory concentrations (MICs) and minimum bactericidal concentrations (MBCs) ranging from 0.78 to 50 mg/mL and 3.125 to 6.25 μL/mL, respectively. These activities can be attributed to binding energies and interactions, such as hydrogen bonding and Van der Waals forces, which were identified in molecular docking studies. The strong attraction between emmotin H, a phenolic compound found in the hydro-ethanolic extract, and the proteins 2ZDQ and 2CAG, which are crucial enzymes in fungi and bacteria, may account for the potent antimicrobial effects of this extract against *Aspergillus niger* and *Candida albicans*. The binding energies between emmotin H and these proteins are −10 and −9.4 kcal/mol, respectively. Undoubtedly, this type of selective molecular interaction is likely to disturb the overall structure and functional stability of these specific proteins, resulting in the suppression of the metabolic processes and development of microorganisms.

## 3. Materials and Methods

### 3.1. Plant Material

*Anethum graveolens* belongs to the *Apiaceae* family, commonly known as dill. The sample under study was collected from a cultivated population in the Errachidia area. Table 13 provides information on the collection location, the component harvested, and the origin. This species was identified at the Laboratory of Botany and Plant Ecology of the Scientific Institute of Rabat, Department of Botany.

### 3.2. Microbiological Materials

In this study, the antimicrobial activity of the essential oil (EO) and extracts of *A. graveolens* seeds was evaluated against five bacterial and five fungal strains. These microorganisms are pathogens commonly encountered in various human diseases (Table 14). These strains were isolated from the hospital environment of the Mohamed V Provincial Hospital, Meknes. All strains were stored in a 20% glycerol stock at −80 °C, rejuvenated in Mueller–Hinton and Sabouraud broths, and subcultured before use.

### 3.3. Quality Control of Plant Material

#### 3.3.1. Moisture Content

The approach used to determine the moisture content complied with the AFNOR standard (NF-V03-402 1985) [69]. A total of 5 g of plant samples were measured using pre-dried and pre-weighed crucibles. The crucibles, which held the plant material, were thereafter placed in an oven and exposed to a temperature ranging from 103 to 105 °C for a duration of 24 h. Subsequently, the samples were subjected to cooling in a desiccator and subsequently weighed. The moisture content was calculated using the Formula (1). The experiment was repeated three times.

(1) $$MC\%=\left(\frac{m_{0}-m_{1}}{m_{0}}\right)\times 100$$

With m_0_ as the initial mass of the plant in (g) and m_1_ as the mass after drying in (g).

The result is expressed as a percentage of dry matter.

#### 3.3.2. Ash Content

The ash content refers to the amount of mineral materials that remain after the organic matter was destroyed by high-temperature incineration in a furnace. A total of 5 g of ground samples were put in a muffle furnace at a temperature of 550 °C until all charred particles were destroyed and whitish ashes were obtained, which had a consistent weight (according to NF ISO 5984) [70]. The organic matter content was determined by employing the subsequent Formula (2):

(2) $$OM\%=\left(\frac{m_{1}-m_{2}}{TE}\right)\times 100$$

OM%: Organic Matter

m_1_: Weight of the capsule and sample before calcination

m_2_: Weight of the capsule and sample after calcination

TE: Test portion

The ash content was calculated as follows (Formula (3)):

**Ash % = 100 − OM%** (3)

#### 3.3.3. Heavy Metal Analysis: Inductively Coupled Plasma Atomic Emission Spectrometry (ICP-AES)

The analysis of the heavy metals in *A. graveolens* seeds was conducted using the technique of Inductively Coupled Plasma Atomic Emission Spectrometry according to the standardized mineralization protocol [71].

This method involved first preparing the sample for analysis in liquid form by mixing 0.1 g of plant powder with 3ml of aqua regia prepared from 1 mL of nitric acid HNO_3_ (99%) and 2 mL of hydrochloric acid HCl (37%), all placed in a reflux setup at 200 °C for two hours to ensure the complete dissolution of residual metal particles. Following the process of chilling and decantation, the liquid remaining above the sediment was gathered. It was then passed through a filter with a pore size of 0.45 µm and brought to a volume of 15 mL by adding distilled water. The concentrations of the heavy metals including arsenic (As), cadmium (Cd), chromium (Cr), iron (Fe), lead (Pb), antimony (Sb), and titanium (Ti) were quantified using an Inductively Coupled Plasma Atomic Emission Spectrometer (Ultima 2 Jobin Yvon) at the laboratory of UATRS (Technical Support Unit for Scientific Research) at CNRST in Rabat.

### 3.4. Phytochemical Screening

This is a qualitative analysis that allows for the identification of primary and secondary metabolites present in *A. graveolens* seeds. These tests were based on the visual observation of color changes, and formation of precipitates and complexes, while other tests included the examination of the samples under UV light. The detection of chemical compound groups was carried out according to protocols described in previous studies [72,73,74,75,76,77]. 

### 3.5. Extraction and Quality Control of Essential Oils

#### 3.5.1. Extraction and Determination of Essential Oil Yields

The extraction of essential oils from *A. graveolens* seeds was carried out by hydrodistillation. A total of 100 g of dried dill seeds were immersed in a 2 L flask containing 1 L of water, topped with a Clevenger apparatus and a ball-type condenser. The mixture was brought to a boil for 3 h. The oils obtained were subsequently dehydrated using anhydrous sodium sulfate (Na_2_SO_4_) and kept in a sealed brown glass container at a temperature of 4 °C until they were ready for use. The extraction yield of the essential oil was quantified as the volume of essential oil obtained per unit mass of plant material (v/m), using Formula (4) [78].

(4) $$\%\;Yield=\left[\frac{V}{m_{0}-{(m}_{0}\times \%\;MC)}\times 10^{4}\right]\mp Standard\;Deviation$$

MC (%): Percentage of moisture in the plant material (moisture content).

m_0_: Mass of distilled plant material

V: Volume of collected essential oil (in mL).

#### 3.5.2. Analysis and Identification of the Chemical Composition of Essential Oils

The chromatographic analysis of essential oils was performed on a gas chromatograph of the Thermo Electron type (Trace GC Ultra) coupled with a mass spectrometer of the Thermo Electron Trace MS system (Thermo Electron: Trace GC Ultra; Polaris Q MS), with fragmentation carried out by electron impact at a 70 eV intensity. The chromatograph is equipped with a DB-5 type column (5% phenyl-methyl-siloxane) (30 m × 0.25 mm × 0.25 μm film thickness) and a flame ionization detector (FID) powered by an H2/Air gas mixture. The column temperature was programmed to increase by 4 °C/min from 50 to 200 °C for 5 min. The injection mode was split (leak ratio: 1/70, flow rate mL/min), and the carrier gas used was nitrogen at a flow rate of 1 mL/min.

The identification of the chemical composition of essential oils was performed by comparing their calculated Kovats indices (IK) with those of Adams and known reference products in the literature [79,80,81]. This was accomplished by conducting a comparison of the retention indices and mass spectra with those found in the mass spectral libraries of the National Institute of Standards and Technologies (NIST). Additionally, the experimental retention indices were compared with those available in the NIST online data collection at https://webbook.nist.gov/chemistry/name-ser/ (accessed on 10 April 2024). The proportions of each component were automatically determined using the total ion count observed by the GC-MS and represented as a percentage composition.

### 3.6. Extraction of Phenolic Compounds

To begin with, in order to isolate the phenolic compounds, two extraction methods were employed: decoction and solid–liquid extraction using the Soxhlet apparatus. The decoction procedure consisted of adding 30 g of the sample to 600 mL of distilled water and heating and bringing the mixture to a boil for one hour at 80 °C. After a five-minute decantation, the mixture was filtered under reduced pressure, and the decocted extract was recovered. This extract was then dried in an oven at 70 °C and subsequently stored in a glass vial.

As for the solid–liquid extraction, it was carried out using the Soxhlet apparatus on two additional samples weighing 30 g each. Two extraction solvents were used: pure water and an ethanol/water mixture in a ratio of 70/30. The extracts were concentrated using a rotary evaporator after several extraction cycles. These extracts were then identified, as indicated in Table 15.

### 3.7. Quantification of Phenolic Compounds

The total phenolic content of the different extracts was determined using the Folin–Ciocalteu method described by Singleton and Rossi [82]. This method is based on the reduction in the basic medium of the mixture of phosphotungstic acid H_3_P(W_3_O_10_)_4_ and phosphomolybdic acid H_3_PMO_12_O_4_ by the oxidizable groups of phenolic compounds present in various *A. graveolens* extracts. The chemical compounds tungsten oxide (W_8_O_23_) and molybdenum oxide (Mo_8_O_3_), which have a blue tint, were examined using colorimetry and optical density measurement. The absorbance measurement was acquired using a UV mini-1240 spectrophotometer configured at a wavelength of 760 nm and then compared to a blank sample, which consisted of a reaction mixture without the extract. A calibration curve was created in parallel, using the same working conditions, with a concentration range of 0.05 to 50 µg/mL of gallic acid as a positive control. The total phenolic content was determined by applying the calibration curve equation (Y = 0.095X + 0.003; R^2^ = 0.998), and the results were expressed in milligrams of gallic acid equivalent per gram of extract (mg GAE/g). The experiment was replicated thrice.

### 3.8. Quantification of Flavonoids

The method by Djeridane and colleagues was used to determine the flavonoid content of our samples using aluminum trichloride as a reagent [83]. The technique is based on the oxidation of flavonoids by this reagent, leading to the formation of a stable yellowish flavonoid–aluminum complex, detectable in the visible range at 433 nm. The flavonoid content in our samples was determined from a calibration range established with increasing concentrations of quercetin ranging from 5 to 30 µg/mL, with the linear regression equation of Y = 0.073X − 0.081 and a determination coefficient R^2^ equal to 0.995. The flavonoid content was expressed in milligrams of quercetin equivalent per gram of extract (mg EQ/g). Each test was repeated three times.

### 3.9. Quantification of Condensed Tannins

The vanillin method was employed to evaluate the concentrations of condensed tannins [84]. During this process, a solution containing vanillin dissolved in methanol at a concentration of 4% *w*/*v* was combined with 20 µL portions of the extracts or a solution of (+)-catechin at a concentration of 2 mg/mL. Subsequently, the mixes were manually agitated. Subsequently, each concentration was transferred to a test tube containing 1.5 milliliters of hydrochloric acid. The reaction mixture was allowed to stand at ambient temperature for a duration of 20 min. The measurement of absorbance was conducted at a wavelength of 499 nm using a UV–visible spectrophotometer, with a reference to a blank sample. The content of condensed tannins in the samples was calculated from the catechin calibration curve used as a standard (Y = 0.7421X + 0.0318; R^2^ = 0.998). The tannin content was expressed in milligrams of catechin equivalent per gram of extract (mg EC/g).

### 3.10. HPLC/UV ESI-MS Analysis of A. graveolens Seed Extracts

The phenolic components of *A. graveolens* in the decoction were analyzed using High-performance liquid Chromatography combined with a Q Exactive Plus mass spectrometer and electrospray ionization as the molecular ionization method (HPLC/UV-ESI-MS). The analysis was performed on an UltiMate 3000 HPLC system (Thermo Fisher Scientific, Sunnyvale, CA, USA) with an autosampler maintained at 5 °C. The HPLC system employed a reverse-phase C18 column with a column temperature of 40 °C (Lichro CART, Lichrospher, Merck, Darmstadt, Germany, 250 × 4 mm, ID 5 µm). The mobile phase was composed of solvent A, which was a mixture of 0.1% formic acid in water (*v*/*v*), and solvent B, which was a mixture of 0.1% formic acid in acetonitrile (*v*/*v*). The gases were eliminated from the mobile phase using the process of sonication. The description of the gradient’s composition may be found in Supplementary Table S1. The rate of flow was 1 milliliter per minute, and the amount injected was 20 µL. After negative electrospray ionization, broadband collision-induced dissociation (bbCID) detection was carried out using a Maxis Impact HD instrument (Bruker Daltonik, Bremen, Germany). The UV detection in the range of 190 to 600 nm and acquisition of three wavelengths from 280 to 320 to 360 nm were performed using a diode array detector L-2455 (Merck-Hitachi, Darmstadt, Germany). The parameters used were a capillary voltage of 3000 V, drying gas temperature of 200 °C, dry gas flow rate of 8 L/min, nebulizer gas pressure of 2 bars, and offset voltage of 500 V. Nitrogen was used as both the nebulizer and desolvation gas. The *m*/*z* range of the MS data was from 100 to 1500.

The process of gathering and examining data was conducted using Thermo Scientific’s Chromeleon 7.2 chromatography data system (CDS). The eluted chemicals were analyzed by studying the mass spectra of the isolated molecules.

### 3.11. Antioxidant Activity

#### 3.11.1. Radical Scavenging Activity by DPPH• Test

The antioxidant activity of the essential oils (EO) and extracts of *A. graveolens* was assessed using the 2,2-diphenyl-1-picrylhydrazyl (DPPH) radical, following the procedure outlined by [85]. A total of 200 µL of *A. graveolens* extract or EO was applied to test tubes containing 100% ethanol. Afterward, 2.8 mL of a solution of DPPH• in ethanol (24 µg/mL, *w*/*v*) was added to the mixture and allowed to incubate for 30 min in the absence of light. The measurement of absorbance was conducted at a wavelength of 515 nm using a UV–Vis spectrophotometer. The experiments were replicated thrice. The reference standard utilized was butylated hydroxytoluene (BHT) at varying concentrations. The results were quantified as a percentage of DPPH• decrease, denoted as AA% (Formula (5)):

(5) $$AA\%=\frac{A_{control}-A_{Sample}}{A_{control}}\times 100$$

AA%: Percentage of antioxidant activity.

A__control_: Absorbance of the solution containing only the DPPH• radical solution.

A__sample_: Absorbance of the test sample solution in the presence of DPPH•.

The 50% inhibitory concentration of the DPPH• free radicals (IC_50_) for BHT or our extracts was determined from a graph of antioxidant activity variation with concentration.

#### 3.11.2. Ferric Reducing Antioxidant Power (FRAP) Method

The capacity of the phenolic extracts from *A. graveolens* to convert ferric iron (Fe^3+^) in the potassium ferricyanide complex to ferrous iron (Fe^2+^) was assessed using the technique outlined by Oyaizu [86]. The experiment consisted of combining 1 mL of the plant extract being investigated with 2.5 mL of a phosphate buffer solution (0.2 M, pH 6.6) and 2.5 mL of a potassium ferricyanide solution (K_3_Fe(CN)_6_ at a concentration of 1%). The resultant mixture was subjected to incubation in a water bath maintained at a temperature of 50 °C for a duration of 20 min. Next, 2.5 mL of a solution containing 10% trichloroacetic acid was introduced to halt the progress of the reaction. The solution was subjected to centrifugation at a speed of 3000 revolutions per minute for a duration of 10 min. Ultimately, 2.5 mL of the liquid remaining after centrifugation from each concentration was combined with 2.5 mL of pure water and 0.5 mL of a solution containing 0.1% FeCl_3_ dissolved in water. The absorbance of the reaction medium was measured at a wavelength of 700 nm using a UV–Vis spectrophotometer. A blank sample was generated in the same way, but distilled water was used instead of the aqueous extract for calibration reasons. The positive control was represented by a solution of a standard antioxidant, BHA (Butylated Hydroxyanisole), for which the absorbance was measured under the same conditions as the samples. All tests were repeated three times. The graph of the variation in reducing power as a function of BHT concentration or our extracts allowed the determination of the concentration corresponding to an absorbance of 0.5 (EC_50_).

#### 3.11.3. Total Antioxidant Capacity

The phosphomolybdenum test described by Khiya et al. [87] was used to screen the total antioxidant capacity of *A. graveolens* extracts. This test is based on the reduction of molybdenum Mo^6+^ (VI) to molybdenum Mo^5+^ (V) in the presence of extracts, leading to the formation of a green complex, phosphate/Mo^5+^ (V) at acidic pH with a maximum absorbance at 695 nm.

In a test tube, a volume of 3 mL of the reactive solution (0.6 M sulfuric acid, 28 mM sodium phosphate, and 4 mM ammonium molybdate) was added to a volume of extract. The tubes were shaken and left in an incubator at 95 °C for 90 min and then normalized to room temperature. After cooling, the absorbance of the solutions was measured at 695 nm. Ascorbic acid was used as a standard. The results were expressed in milligrams of ascorbic acid equivalents per gram of extract (mg AAE/g).

### 3.12. Antimicrobial Activity

The Minimum Inhibitory Concentration (MIC) represents the lowest concentration of essential oil or extract capable of completely inhibiting the growth of a microorganism. The determination of the MIC was performed using the microdilution method [88]. Initially, a stock solution of essential oil, prepared in 10% DMSO, was subjected to a series of dilutions to obtain concentrations ranging from 5 to 0.93 × 10^−2^ mg/mL for the essential oil. As for the extracts, a stock solution was prepared beforehand and then diluted to achieve the desired concentrations, expressed in µg/mL. The dilutions of essential oil and extracts were carried out in a Mueller–Hinton broth for bacteria and Sabouraud broth for fungi, with a final volume of 100 µL for each concentration.

Afterwards, 100 µL of the microbial inoculum, with a final concentration of 10^6^ CFU/mL for bacteria and 10^4^ CFU/mL for fungus, were added to the various concentrations of the dilution series. Following a 24 h incubation period at a temperature of 37 °C, 10 µL of resazurin was introduced into each well as a means of detecting bacterial proliferation. After a further incubation period of 2 h at a temperature of 37 °C, the presence of microbial growth was indicated by a shift in hue from violet to pink. The minimum inhibitory concentration (MIC) is defined as the lowest concentration of a substance that effectively blocks the observed color shift of resazurin. The 11th and 12th wells in each series were designated as the growth control and sterility control, respectively. This technique was replicated twice for both the oil and extracts.

To determine the Minimum Bactericidal Concentration (MBC) or Minimum Fungicidal Concentration (MFC), 10 µL was taken from each well without visible growth and inoculated on Mueller–Hinton agar (MH) for bacteria or Sabouraud agar for fungi. The plates were incubated for 24 h at 37 °C. The MBC and MFC were defined as the lowest concentration of the samples analyzed, which resulted in a 99.99% reduction in CFU/mL compared to the control. The ratio of MBC/MIC or MFC/MIC was calculated to assess the antimicrobial power. A ratio less than 4 indicated a bactericidal/fungicidal effect of the essential oil, while a ratio greater than 4 indicated a bacteriostatic/fungistatic effect of the sample.

### 3.13. PASS, ADMET, Pro-Tox, and Prediction of the Efficacy of Potentially Active Compounds Isolated from the Essential Oil and Aqueous Extract of A. graveolens Seeds

The main constituents of the essential oil (EO) and aqueous extract derived from *A. graveolens* seeds examined were selected for ADMET (absorption, distribution, metabolism, excretion, and toxicity) tests and PASS prediction tests. To choose the SMILES format of these chemical substances, ChemBioDraw (PerkinElmer Informatics, Waltham, MA, USA, v13.0) [56] was used. Subsequently, simulations were conducted using the PASS-Way2Drug online prediction tool [57], as well as the online tools SwissADME and pkCSM for ADMET prediction [58]. The potential activity (Pa) and probable inactivity (Pi) of “drug-like” substances were designated by the PASS acronym [89].

ProTox II, a useful tool created specifically for this purpose, was used to examine toxicity levels and gather relevant data on several toxicological parameters, including LD50 and the toxicity class [90]. The ADMET software, consisting of SwissADME, pkCSM, and ProTox II, was employed to assess the chosen ligands. This allowed for the prediction of their physicochemical properties, lipophilicity, water solubility, pharmacokinetics, drug-likeness, medicinal chemistry, and toxicological features. The utilization of these methodologies and analytical instruments enabled reliable observations on the possible therapeutic uses and negative consequences linked to the primary chemical constituents found in the EO and aqueous extract of *A. graveolens* seeds being studied.

### 3.14. Molecular Docking

The protein targets’ three-dimensional structures, as indicated in Table 16, were acquired from the Protein Data Bank RCSB, which may be accessed at the URL https://www.rcsb.org/ (accessed on 10 April 2024). The visualization of protein structure was conducted using UCSF Chimera. The protein structures were constructed as suitable docking targets using Autodock Tools (version 1.5.6, The Scripps Research Institute, La Jolla, CA, USA). Before analysis, the protein structures underwent preprocessing steps, including the removal of water molecules, heteroatoms (hetatm), unwanted protein chains, and co-crystallized ligands. Subsequently, polar hydrogen atoms and Gesteiger charges were added, and the resulting structures were converted to pdbqt format for further analysis.

The three-dimensional structures of the sixteen compounds were represented using their respective structures, collected from PubChem, and then subjected to structural energy minimization. The ligands were then processed using the Prescription Virtual Screening Python script (AutoDock Vina 1.2.0), where their SDF files were converted into pdbqt formats.

The compound docking process utilized the scoring function of AutoDock Vina. After selecting the protein and ligand molecules for docking in the Vina control, a grid box was overlaid onto the protein structure. The grid box size could be adjusted based on the chosen active site residues before launching the AutoDock Vina program for the docking process. The search space was bounded by the grid box, whose size and position were determined by coordinates to precisely align with the active binding site (Table 16). The resulting data for docked molecules, expressed in free binding energy values, were recorded, and the analysis of ligand-protein binding features was conducted using PyMOL.

Subsequently, we proceeded with redocking, a method representing a reliable approach to validate the molecular docking process. This method involves separating the crystallized ligand from the protein, followed by a new docking analysis in the same region where the ligand was initially located. This approach allows us to accurately assess whether the docked ligand effectively overlaps with the crystallized ligand by closely examining the RMSD parameter value using specialized software such as PyMOL 2.5.0. In summary, redocking offers rigorous validation of the molecular docking process by confirming the consistency of the results obtained.

### 3.15. Molecular Dynamics Simulation

The GROMACS 2019.3 software was employed to perform molecular dynamics simulations in order to evaluate the stability of the protein–ligand complex [91]. The protein was subjected to the all-atom CHARMM36 force field, and the topologies of the ligands were generated using the CGenFF service. The systems were rendered neutral by introducing ions to counterbalance the overall charges after the immersion of all complexes in a rectangular container filled with TIP3P water molecules. The steepest descent strategy was used to achieve both the least energy and maximum force by selecting a force threshold (Fmax) of 1000 kJ/mol/nm. The ensembles were maintained at constant NVT (number of atoms, volume, and temperature) and NPT (number of atoms, pressure, and temperature) for molecular dynamics simulation investigations. Subsequently, every molecule underwent molecular dynamics simulations lasting 100 ns. Analyzed output trajectories yielded a comprehensive understanding of protein behavior.

### 3.16. Statistical Analysis

The outcomes are presented as the average, and the measure of variability is known as the standard deviation. The data were evaluated using GraphPad Prism 8 software (version 8.0.2, San Diego, CA, USA) by the use of a one-way ANOVA test and Tukey’s post-test. Significance was attributed to P values below 0.05. The relationship between the phenolic compounds and antioxidant activity content was assessed using the Pearson correlation coefficient. A difference was considered statistically significant when the P-value was less than 0.05.

## 4. Conclusions

This study emphasized the abundance of phytochemicals in the seeds of *Anethum graveolens*. The essential oil was analyzed by GC-MS, which showed that (E)-anethole was the most abundant compound at 38.13%, followed by estragole at 29.32%, fenchone at 17.21%, and α-pinene at 7.37%. The seeds furthermore possessed secondary metabolites, including tannins, flavonoids, sterols, triterpenes, and mucilage.

This quantitative study revealed substantial concentrations of polyphenols, flavonoids, and condensed tannins. The use of HPLC/UV-ESI-MS analysis enabled the detection and identification of 38 prominent compounds, such as umbelliferone (12.35%), hydroxyflavone (11.23%), rosmanol (8.95%), biotin (8.36%), Emmotin H (4.91%), and coumarin (4.21%).

The essential oils and extracts exhibited significant antioxidant activity as demonstrated by the DPPH˙, FRAP, and CAT tests. They were able to effectively neutralize free radicals and bind metal ions. The antimicrobial tests demonstrated that the essential oil exhibited more efficacy compared to the aqueous, ethanolic, and decoction extracts against the majority of the bacteria tested. This enhanced effectiveness can be attributed to the synergistic effects of its primary constituents.

The docking and molecular dynamics simulations validated substantial connections and enduring stability between certain bioactive chemicals and diverse target proteins.

The findings indicate that *A. graveolens* exhibits a significant therapeutic promise for a range of human diseases and has the ability to serve as a natural preservative in the food business.

## Figures and Tables

**Table 1:** Analysis and quality control of *A. graveolens* seeds.

MC (%)	pH	Ash (%)	Organic Matter (%)
15.80	5.2	15.03	84.97

**Table 2:** Heavy metal concentration (mg/L) and maximum limit FAO/WHO (2009).

Element	Heavy Metal Content (mg/L)	Maximum Limit
Chromium (Cr)	0.0008	2
Antimony (Sb)	0.0134	1
Lead (Pb)	0.0034	3
Cadmium (Cd)	<0.0001	0.3
Iron (Fe)	0.5858	20
Titanium (Ti)	0.0082	-

**Table 3:** Essential Oil Yield of *A. graveolens*.

Properties	Yield (%)	Color	Odor	Density (g/mL)
Results	2.73 ± 0.12	Yellowish	Aromatic	0.9362 ± 0.0032

**Table 4:** Chemical Composition of *Anethum graveolens* Essential Oil.

N°	Compounds	KI *	RA%	Formula
**1**	α-Pinene	939	7.37	C_10_H_16_
**2**	Camphene	954	0.08	C_10_H_16_
**3**	Sabinene	975	0.13	C_10_H_16_
**4**	β-Pinene	979	0.35	C_10_H_16_
**5**	Myrcene	990	0.76	C_10_H_16_
**6**	α-Phellandrene	1002	0.17	C_10_H_16_
**7**	α-Terpinene	1017	0.02	C_10_H_16_
**8**	Limonene	1029	1.24	C_10_H_16_
**9**	β-Phellandrene	1029	0.37	C_10_H_16_
**10**	Z-β-Ocimene	1037	0.16	C_10_H_16_
**11**	1,8-Cineole	1031	0.11	C_10_H_18_O
**12**	γ-Terpinene	1059	0.66	C_10_H_16_
**13**	Terpinolene	1088	0.05	C_10_H_16_
**14**	**Fenchone**	**1086**	**17.21**	C_10_H_16_O
**15**	Linalool	1096	2.68	C_10_H_18_O
**16**	trans-Pinene hydrate	1122	0.03	C_10_H_18_O
**17**	Camphor	1146	0.35	C_10_H_16_O
**18**	Terpinen-4-ol	1177	0.13	C_10_H_18_O
**19**	**Estragole**	**1196**	**29.32**	C_10_H_12_O
**20**	Carvone	1243	0.01	C_10_H_14_O
**21**	Z-Anethole	1252	0.06	C_10_H_12_O
**22**	ρ-Anis aldehyde	1250	0.05	C_8_H_8_O_2_
**23**	**E-Anethole**	**1284**	**38.13**	C_10_H_12_O
**24**	α-Copaene	1376	0.02	C_15_H_24_
**25**	Z-Isoeugenol	1407	0.02	C_10_H_12_O_2_
**26**	Methyleugenol	1403	0.34	C_11_H_14_O_2_
**27**	Germacrene D	1481	0.09	C_15_H_24_
**28**	δ-Cadinene	1523	0.03	C_15_H_24_
**29**	E-Methylisoeugenol	1492	0.06	C_11_H_14_O_2_
**% Hydrogenated Monoterpenes**				**11.36**
**% Oxygenated Monoterpenes**				**20.57**
**% Phenylpropanoids**				**67.93**
**% Hydrogenated Sesquiterpenes**				**0.14**
**Total**				**100**

* RA: Relative abundance (%); KI: Kovats index.

**Table 5:** Results of phytochemical tests conducted on extracts of *A. graveolens* seeds.

Chemical Group		*A. graveolens*
**Polysaccharide**		starch
**Reducing sugars**		+
**Protein**	**Biuret reaction**	+
	**Xanthoprotein reaction**	+
**Lipids (Lieberman Burchard reaction)**		+++
**Tannins**		+++
**Catechic tannins**		++
**Gallic tannins**		++
**Flavonoids**		++
**Leucoanthocyanins**		++
**Saponosides**		-
**Alkaloids**		-
**Reducing Compounds**		++
**Oses and holosides**		++
**Mucilages**		++
**Sterols and triterpenes**		++

+++ Very abundant; ++: abundant; + weak; - absent.

**Table 6:** List of compounds identified by HPLC/UV-ESI-MS in the decoction E (0) of *A. graveolens* seeds.

N°	RT	Area (%)	Molecules	Structure	Classes	Exact Weights	[M-H]^−^ (*m*/*z*)	[M+H]^+^ (*m*/*z*)	Fragment Ions (*m*/*z*)
1	4.05	1.8	Medioresinol	C_21_H_23_O_7_	Flavonoid	388	387		207-179
2	4.23	1.75	Caffeic acid	C_9_H_8_O_4_	Phenolic acid	180		181	181-163-145-139-114
3	4.33	2.47	Cinnamic acid	C_9_H_8_O_2_	Phenolic acid	148	147		119-103-93
4	4.51	1.21	Scopoletin	C_10_H_8_O_4_	Coumarin	192	191		176-148-104
5	4.75	3.58	Pimelic acid	C_7_H_12_O_4_	Fatty acid	160	159		115-97
6	5.34	0.53	Retusin	C_19_H_18_O_7_	Flavonoid	358	357		357-342-327-312-297
7	8.39	0.60	Syringic acid	C_9_H_10_O_5_	Phenolic acid	198	197		179-135-123
8	8.53	1.30	Carnosic acid	C_20_H_27_O_4_	Diterpene	332		333	333-287
9	8.96	12.35	Umbelliferone	C_9_H_6_O_3_	Coumarin	162	161		133-106
10	9.00	8.36	Biotin	C_10_H_16_N_2_O_3_S	Vitamin	244		243	297-228-174-130
11	9.24	4.21	Coumarin	C_9_H_6_O_2_	Coumarin	146		147	147-103
12	9.48	1.34	Kaempferide	C_16_H_12_O_6_	Flavonoid	300	299		217-149-107
13	9.91	11.23	3-Hydroxyflavone	C_15_H_10_O_3_	Flavonoid	238	237		237-135-101
14	10.15	8.95	Rosmanol	C_20_H_26_O_5_	Polyphenol	346	345		243-197-147
15	10.78	3.40	Homovanillic acid	C_9_H_10_O_4_	Other	182	181		137-123-108
16	11.08	3.35	kaempferol	C_15_H_10_O_6_	Flavonoid	286	285		201-165-151-117
17	11.37	3.56	Methyl rosmarinate	C_19_H_18_O_8_	Polyphenol	374		375	375-181-137
18	11.55	0.98	Chlorogenic acid	C_16_H_18_O_9_	Phenolic acid	354	353		191-179
19	12.33	0.55	Apigenin 7-rhamnoside	C_21_H_20_O_9_	Flavonoid	416		417	417-271-243-229
20	12.71	1.02	Quercitine 7-glucuronide	C_21_H_18_O_13_	Flavonoid	478	477		431-301-175
21	12.99	0.42	Epicatechin gallate	C_22_H_18_O_10_	Polyphenol	442	441		289-169-125
22	13.72	1.26	Rhamnetin	C_16_H_12_O_7_	Flavonoid	316	315		300-179-165-151
23	13.92	0.42	Riboflavin	C_17_H_20_N_4_O_6_	Vitamin	376	375		375-255-243-241
24	14.11	1.29	Quercetin-3-O-galactoside	C_21_H_20_O_12_	Flavonoid	464		465	465-427-303-91
25	14.80	1.29	Quercetin 3-O-rhamnoside	C_21_H_20_O_11_	Flavonoid	448	447		301-300-284
26	15.25	1.67	δ-tocopherol	C_27_H_46_O_2_	Vitamin	402	401		385-135
27	15.73	3.85	trans-caftaric acid	C_13_H_12_O_9_	Phenolic acid	312	311		249-203-179-149
28	16.10	1.03	3-Feruloylquinic acid	C_17_H_20_O_9_	Phenolic acid	368	367		293-209-193-173
29	16.60	0.56	Quercetin 3,3′-dimethyl ether	C_17_H_14_O_7_	Flavonoid	330	329		314-301-285-270
30	17.35	1.62	Rosmarinic acid	C_18_H_16_O_8_	Phenolic acid	360		361	181-163-145-135-117
31	19.06	1.23	Apigenin	C_15_H_10_O_5_	Flavonoid	270	269		227-159-121-105
32	20.26	0.78	Apigenin-7-glucoside	C_21_H_20_O_10_	Flavonoid	432	431		431-385-268-240-151-107
33	20.41	0.80	Folic acid	C_19_H_19_N_7_O_6_	Vitamin	441			311-267-224-175
35	21.43	4.91	Emmotin H	C_15_H_16_O_3_	Sesquiterpenoid	244	243		243-228-200-184
36	23.74	0.47	caffeoyl-feruloyltartaric acid	C_23_H_20_O_12_	Phenolic acid	488	487		487-443-293
37	25.35	3.37	1-Caffeoyl-beta-D-glucose	C_15_H_18_O_9_	Polyphenol	342	341		341-179-135
38	25.76	0.45	Ascorbyl palmitate	C_22_H_38_O_7_	Other	414		415	415-371-115

**Table 7:** Correlation coefficients (R^2^) between polyphenol (PC),flavonoid (FC), tannin content (TC) and antioxidant activity of *A. graveolens* extract.

	PC	FC	TC	DPPH	FRAP	CAT
**PC**	1	0.907	0.994	0.970	0.990	0.762
**FC**	0.907	1	0.856	0.982	0.957	0.964
**TC**	0.994	0.856	1	0.938	0.969	0.969
**DPPH**	0.970	0.982	0.938	1	0.995	0.896
**FRAP**	0.990	0.957	0.969	0.995	1	0.845
**CAT**	0.762	0.964	0.686	0.896	0.845	1

**Table 8:** MICs and MBCs (mg/mL) of *A. graveolens* EO and extracts (ethanolic, aqueous, and decocted).

Bacterial Strains	E (0)			E (1)			E (2)			EO		
	MIC	MBC	MBC/MIC	MIC	MBC	MBC/MIC	MIC	MBC	MBC/MIC	MIC	MBC	MBC/MIC
*E. cloacae*	50	50	1	>50	>50	-	50	50	1	25	25	1
*K. pneumoniae*	50	50	1	50	50	1	50	50	1	25	25	1
*E. coli*	>50	>50	-	>50	>50	-	>50	>50	-	25	25	1
*S. aureus*	50	50	1	50	50	1	50	50	1	25	50	2
*S. epidermidis*	>50	>50	-	>50	>50	-	>50	>50	-	50	50	1

**Table 9:** MIC and MFC of *A. graveolens* EO and extracts (ethanolic, aqueous, and decocted).

Fungal Strains	E (0)			E (1)			E (2)			EO		
	MIC	MFC	MFC/MIC	MIC	MFC	MFC/MIC	MIC	MFC	MFC/MIC	MIC	MFC	MFC/MIC
*C. albicans*	>50	>50	-	50	50	1	50	50	1	3.125	6.25	2
*C. dubliniensis*	>50	>50	-	6.25	6.25	1	12.5	12.5	1	3.125	3.125	1
*C. tropicalis*	>50	>50	-	12.5	12.5	1	25	25	1	6.25	6.25	1
*C.parapsilosis*	>50	>50	-	12.5	12.5	1	12.5	12.5	1	6.25	6.25	1
*A. niger*	>50	>50	-	0.78	0.78	1	25	25	1	3.125	3.125	1

**Table 10:** In Silico Analysis of PASS, ADME of Compounds from the Essential Oil, and Decoction of *A. graveolens* Seeds.

Prediction	Parameters	EO 1	EO 2	EO 3	EO 4	D 1	D 2	D 3	D 4	D 5	D 6	D 7	D 8	D 9	D 10	D 11	D 12
**PASS Prediction (Pa/Pi)**																	
Antioxidant	Antioxidant	0.337/0.018			0.563/0.005	0.641/0.004	0.551/0.005	0.150/0.103	0.286/0.026	0.389/0.013	0.711/0.004	0.260/0.033	0.483/0.007	0.321/0.020	0.751/0.004	0.856/0.003	0.337/0.018
Antimicrobial	Antifungal	0.425/0.045	0.267/0.097	0.439/0.042	0.519/0.027	0.367/0.058	0.639/0.014	0.494/0.032	0.504/0.030	0.440/0.041	0.544/0.024	0.363/0.059	0.474/0.035	0.292/0.084	0.717/0.009	0.495/0.031	0.425/0.045
	Antibacterial	0.264/0.075	0.219/0.102	0.326/0.051	0.398/0.030	0.331/0.049	0.569/0.011	0.357/0.041	0.301/0.060	0.344/0.045	0.303/0.059	0.292/0.063	0.218/0.102	0.216/0.104	0.587/0.010	0.395/0.031	0.264/0.075
**ADME Prediction**																	
Physiochemical Properties	TPSA (Å^2^)	9.23	9.23	17.07	0.00	50.44	50.44	86.99	103.73	54.37	30.21	161.59	74.60	133.52	66.76	156.91	111.13
	Molar Refractivity	47.83	47.04	45.64	45.22	44.51	69.94	93.99	69.34	70.37	42.48	70.60	39.31	95.72	46.50	79.13	76.01
Drug Likeness Prediction	Bioavailability Score	0.55	0.55	0.55	0.55	0.55	0.55	0.55	0.56	0.55	0.55	0.11	0.85	0.55	0.85	0.55	0.55
	Synthetic accessibility	1.47	1.28	3.22	4.44	2.56	2.93	5.07	3.38	3.03	2.74	3.45	1.37	3.52	1.49	4.47	3.14
Absorption Parameters Prediction	Water solubility	−2.936	−2.874	−3.097	−3.733	−2.131	−3.683	−3.606	−2	−3.366	−1.517	−2.541	−1.088	−3.17	−2.453	−1.869	−3.04
	Caco2 permeability	1.669	1.41	1.501	1.38	1.206	1.263	1.015	0.698	1.267	1.649	−0.801	0.598	−0.562	0.265	0.05	0.032
	Intestinal absorption (human)	95.592	94.536	95.813	96.041	94.551	94.776	93.407	71.182	96.41	97.344	9.399	72.877	64.776	86.286	50.517	74.29
	Skin Permeability	−1.139	−1.739	−1.872	−1.827	−2.6	−2.775	−2.772	−2.727	−2.93	−1.921	−2.735	−2.735	−2.735	−2.722	−2.74	−2.735
	P-glycoprotein substrate	No	No	No	No	No	Yes	Yes	No	No	No	No	No	Yes	Yes	No	Yes
	P-glycoprotein I inhibitor	No	No	No	No	No	No	Yes	No	No	No	No	No	No	No	No	No
	P-glycoprotein II inhibitor	No	No	No	No	No	No	No	No	No	No	No	No	No	No	No	No
Distribution Parameters Prediction	VDss (human)	0.343	0.401	0.341	0.667	0.032	0.214	0.653	−0.933	0.161	−0.143	−0.919	−1.429	0.75	−1.533	0.141	1.274
	Fraction unbound (human)	0.266	0.213	0.423	0.425	0.432	0.151	0.109	0.58	0.3	0.367	0.472	0.543	0.229	0.467	0.609	0.178
	BBB permeability	0.529	0.601	0.624	0.791	−0.278	0.462	−0.581	−0.679	0.291	−0.007	−1.233	−0.21	−1.454	−0.312	−1.081	−0.939
	CNS permeability	−1.659	−1.74	−1.988	−2.201	−2.741	−1.733	−2.101	−3.541	−2.784	−1.926	−3.93	−3.042	−3.358	−2.723	−3.982	−2.228
Metabolism Parameters Prediction	CYP2D6 substrate	No	No	No	No	No	No	No	No	No	No	No	No	No	No	No	No
	CYP3A4 substrate	No	No	No	No	No	Yes	No	No	No	No	No	No	Yes	No	No	No
	CYP1A2 inhibitor	Yes	Yes	No	No	Yes	Yes	No	No	Yes	Yes	No	No	No	No	No	Yes
	CYP2C19 inhibitor	No	No	No	No	No	Yes	No	No	No	No	No	No	No	No	No	No
	CYP2C9 inhibitor	No	No	No	No	No	No	No	No	No	No	No	No	No	No	No	No
	CYP2D6 inhibitor	No	No	No	No	No	No	No	No	No	No	No	No	No	No	No	No
	CYP3A4 inhibitor	No	No	No	No	No	No	No	No	No	No	No	No	No	No	No	No
Excretion	Total Clearance	0.268	0.332	0.085	0.043	0.706	0.233	0.289	0.368	0.19	0.97	0.449	0.565	0.187	0.246	0.059	0.477
	Renal OCT2 substrate	No	No	No	No	No	No	No	No	No	No	No	No	No	No	No	No

EO1: E-Anethole; EO2: Estragole; EO3: Fenchone; EO4: α-Pinene; D1: Umbelliferone; D2: 3-hydroxyflavone; D3: Rosmanol; D4: Biotine; D5: Emmotin H; D6: Coumarin; D7: trans-Caftaric acid; D8: pimelic acid; D9: Methyl rosmarinate; D10: Homovanillic Acid; D11: 1-Caffeoyl-beta-D-glucose; D12: Kaempferol.

**Table 11:** In silico analysis of the predictive toxicity (ProTox II) of Compounds from the Essential Oil and Decoction of *A. graveolens* Seeds.

Compounds	AMES Toxicity	hERG I Inhibitor	hERG II Inhibitor	Skin Sensitization	Minnow Toxicity	LD_50_ (mg/kg)	Predicted Toxicity Class	Hepatotoxicity	Carcinogenicity	Immunotoxicity	Mutagenicity	Cytotoxicity
**EO1**	No	No		Yes	0.869	150	3	Inactive	Active (0.57)	Inactive		
**EO2**	Yes			Yes	1.398	1230	4		Active (0.51)			
**EO3**	No			Yes	1.366	775	4		Inactive			
**EO4**	No			No	1.159	3700	5		Inactive			
**D1**	No	No	No	No	1.714	10,000	6	Inactive	Active (0.64)	Inactive	Inactive	Inactive
**D2**	Yes		No		1.205	2500	5	Inactive	Inactive	Inactive		Inactive
**D3**	No		Yes		0.285	450	4	Inactive	Inactive	Active (0.95)		Inactive
**D4**	No		No		2.183	4000	5	Active (0.41)	Inactive	Inactive		Inactive
**D5**	No		No		1.329	1600	4	Inactive	Inactive	Inactive		Inactive
**D6**	No		No		1.555	196	3	Inactive	Active (0.83)	Inactive		Active (0.55)
**D7**	No		No		4.872	3800	5	Inactive	Inactive	Active (0.97)		Inactive
**D8**	No		No		2.006	900	4	Inactive	Inactive	Inactive		Inactive
**D9**	No		No		1.92	5000	5	Inactive	Inactive	Active (0.93)		Inactive
**D10**	No		No		2.106	1123	4	Inactive	Inactive	Inactive		Inactive
**D11**	No		No		5.989	5000	5	Inactive	Inactive	Active (0.95)		Inactive
**D12**	No		No		2.885	3919	5	Inactive	Inactive	Inactive		Inactive

EO1: E-Anethole; EO2: Estragole; EO3: Fenchone; EO4: α-Pinene; D1: Umbelliferone; D2: 3-hydroxyflavone; D3: Rosmanol; D4: Biotine; D5: Emmotin H; D6: Coumarin; D7: trans-Caftaric acid; D8: pimelic acid; D9: Methyl rosmarinate; D10: Homovanillic Acid; D11: 1-Caffeoyl-beta-D-glucose; D12: Kaempferol.

**Table 12:** Details of the Binding Affinities of Antimicrobial and Antioxidant Target Proteins with Selected Ligands.

Ligands\Targets	1JZQ	1KZN	2CAG	2VEG	2ZDQ	3SRW	3UDI	4URN	2CDU	1OG5	3NRZ
**EO1**	−5.1	−5.6	−7.1	−4.8	−7.1	−5.8	−4.7	−5.5	−5.6	−5.4	−7.5
**EO2**	−5.2	−5.6	−6.7	−4.5	−7.0	−5.5	−4.6	−5.3	−5.5	−5.3	−7.0
**EO3**	−5.6	−4.8	−6.5	−4.6	−6.6	−5.9	−5.0	−4.7	−5.3	−6.0	−6.2
**EO4**	−5.5	−4.6	−6.0	−4.6	−6.2	−5.6	−4.5	−4.6	−5.0	−5.5	−5.1
**D1**	−6.0	−6.7	−8.1	−5.8	−8.2	−6.4	−5.8	−6.2	−6.4	−6.3	−8.8
**D2**	−7.6	−7.7	−9.4	−7.0	−8.4	−8.1	−7.4	−7.5	−7.5	−7.9	−8.8
**D3**	−8.2	−8.0	−7.2	−7.0	−6.5	−8.6	−8.2	−7.7	−7.3	−8.9	−6.2
**D4**	−6.3	−6.2	−7.5	−5.3	−7.3	−7.0	−6.0	−6.2	−6.1	−7.1	−7.0
**D5**	−7.5	−7.5	−9.0	−6.2	−10.0	−8.0	−7.6	−6.3	−7.6	−8.0	−6.2
**D6**	−5.8	−6.6	−7.7	−5.5	−7.9	−6.3	−5.6	−5.8	−6.4	−6.6	−8.1
**D7**	−6.8	−7.3	−8.9	−6.8	−9.1	−7.8	−7.2	−6.5	−7.9	−7.3	−7.6
**D8**	−4.9	−5.2	−5.9	−4.5	−5.8	−4.9	−4.9	−4.9	−5.1	−5.4	−6.6
**D9**	−7.8	−8.4	−10.2	−7.8	−9.2	−8.4	−8.3	−7.3	−8.0	−8.4	−9.8
**D10**	−5.5	−6.1	−7.1	−5.4	−7.0	−6.0	−5.5	−5.5	−6.1	−6.0	−7.4
**D11**	−7.6	−7.6	−9.5	−6.7	−9.8	−8.0	−7.4	−7.0	−7.7	−7.9	−10.0
**D12**	−8.1	−8.2	−10.0	−6.6	−9.0	−8.5	−8.3	−7.9	−7.7	−8.6	−7.8

EO1: E-Anethole; EO2: Estragole; EO3: Fenchone; EO4: α-Pinene; D1: Umbelliferone; D2: 3-hydroxyflavone; D3: Rosmanol; D4: Biotine; D5: Emmotin H; D6: Coumarin; D7: trans-Caftaric acid; D8: pimelic acid; D9: Methyl rosmarinate; D10: Homovanillic Acid; D11: 1-Caffeoyl-beta-D-glucose; D12: Kaempferol.

**Table 13:** Harvesting Site, Parts Used, Habitat, and Harvesting Season for *A. graveolens*.

Scientific Name	Part Collected	Harvesting Area					
		Region	Location	Latitude (x)	Longitude (y)	Altitude (m)	Harvest Period
*Anethum graveolens*	Seeds	Errachidia	Annif	31°06′54″ N	5°09′38″ W	905	June 2022

**Table 14:** List of tested bacterial and fungal strains used for antimicrobial tests.

Strains			Abbreviations
**Bacteria**	**Gram-negative** **bacilli**	*Enterobacter cloacae*	*E. cloacae*
		*Klebsiella pneumoniae*	*K. pneumoniae*
		*Escherichia coli*	*E. coli*
	**Gram-positive cocci**	*Staphylococcus aureus*	*S. aureus*
		*Staphylococcus epidermidis*	*S. epidermidis*
**Fungi**	**Yeasts**	*Candida albicans*	*C. albicans*
		*Candida dubliniensis*	*C. dubliniensis*
		*Candida tropicalis*	*C. tropicalis*
		*Candida parapsilosis*	*C. parapsilosis*
	**Mold**	*Aspergillus niger*	*A. niger*

**Table 15:** Coding of *A. graveolens* extracts.

Extraction Methods	Solvents	Coding
**Decoction**	Water	E (0)
**Soxhlet**	Ethanol/water (70/30; *v*/*v*)	E (1)
	Water	E (2)

**Table 16:** Protein targets and molecular docking parameters.

Protein	PDB ID	Grid Box Center Coordinates	Grid Box Size
Isoleucyl-tRNA synthetase	1JZQ	center_x = −27.803	size_x = 34
		center_y = 6.619	size_y = 22
		center_z = −28.722	size_z = 21
DNA gyrase	1KZN	center_x = 18.325	size_x = 23
		center_y = 30.783	size_y = 38
		center_z = 36.762	size_z = 38
Catalase compound II	2CAG	center_x = 60.017	size_x = 28
		center_y = 14.760	size_y = 22
		center_z = 15.935	size_z = 34
Dihydropteroate synthase	2VEG	center_x = 31.404	size_x = 24
		center_y = 48.530	size_y = 24
		center_z = 0.204	size_z = 23
D-Alanine ligase	2ZDQ	center_x = 47.378	size_x = 21
		center_y = 12.782	size_y = 26
		center_z = 5.730	size_z = 32
Dihydrofolate reductase	3SRW	center_x = -4.701	size_x = 20
		center_y = -31.536	size_y = 26
		center_z = 6.341	size_z = 26
Penicillin-binding protein 1a PBP1a	3UDI	center_x = 34.198	size_x = 24
		center_y = −1.249	size_y = 22
		center_z = 12.715	size_z = 28
Crystal structure of Staph ParE 24 kDa	4URN	center_x = -31.684	size_x = 28
		center_y = 8.021	size_y = 40
		center_z = −4.598	size_z = 42
NADPH oxidase	2CDU	center_x = 18.26	size_x = 22
		center_y = −6.35	size_y = 24
		center_z = −1.53	size_z = 28
Cytochrome P450 2C9	1OG5	center_x = −20.236	size_x = 21
		center_y = 86.761	size_y = 23
		center_z = 38.657	size_z = 20
Xanthine dehydrogenase/oxidase	3NRZ	center_x = 37.526	size_x = 20
		center_y = 19.929	size_y = 20
		center_z = 17.643	size_z = 20

## Data Availability

Data are contained within this article.

## Supplementary Materials

The following supporting information can be downloaded at https://www.mdpi.com/article/10.3390/ph17070862/s1, Figure S1. GC-MS chromatogram of the essential oil from *A. graveolens*; Figure S2. Structures of the main compounds identified in *A. graveolens* EO; Figure S3. HPLC chromatogram of compounds from the decocted extract of *A. graveolens*; Figure S4. Structures of the majority of compounds identified in extract E (0) of *A. graveolens*; Table S1. Gradient mobile phase elution.
