# Supplementary material for: Phytochemical Analysis and Evaluation of Antioxidant and Antimicrobial Properties of Essential Oils and Seed Extracts of Anethum graveolens from Southern Morocco: In Vitro and In Silico Approach for a Natural Alternative to Synthetic Preservatives
Source: Pharmaceuticals (Basel). 2024 Jul 1;17(7):862. doi: 10.3390/ph17070862 (PMC11280095; doi:10.3390/ph17070862)
Supplement: Supplementary file 1 [file pharmaceuticals-17-00862-s001.zip › pharmaceuticals-3060454-supplementary.pdf]

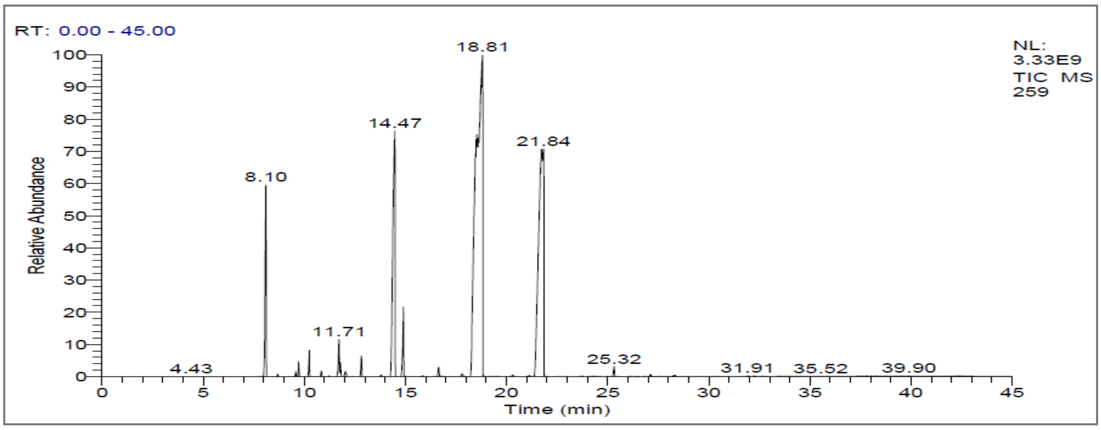

Figure S1. GC-MS chromatogram of the essential oil from *A. graveolens*.

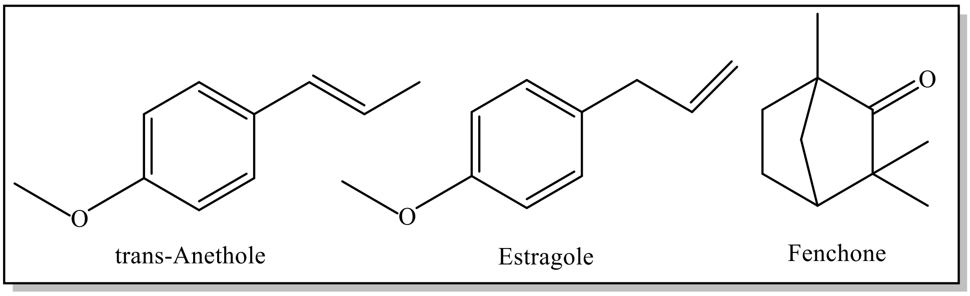

Figure S2. Structures of the main compounds identified in *A. graveolens* EO

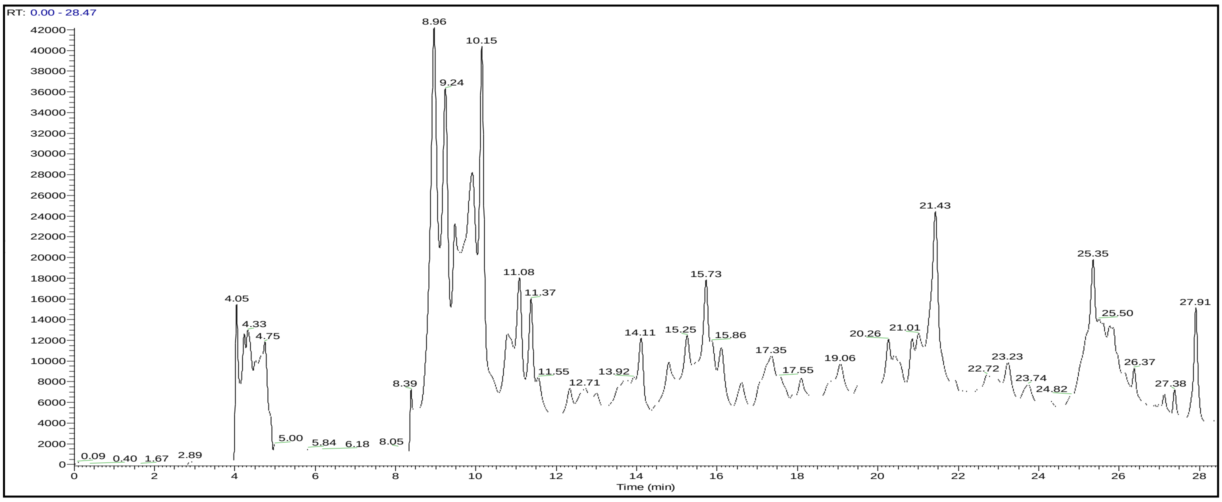

Figure S3. HPLC chromatogram of compounds from the decocted extract of *A. graveolens*.

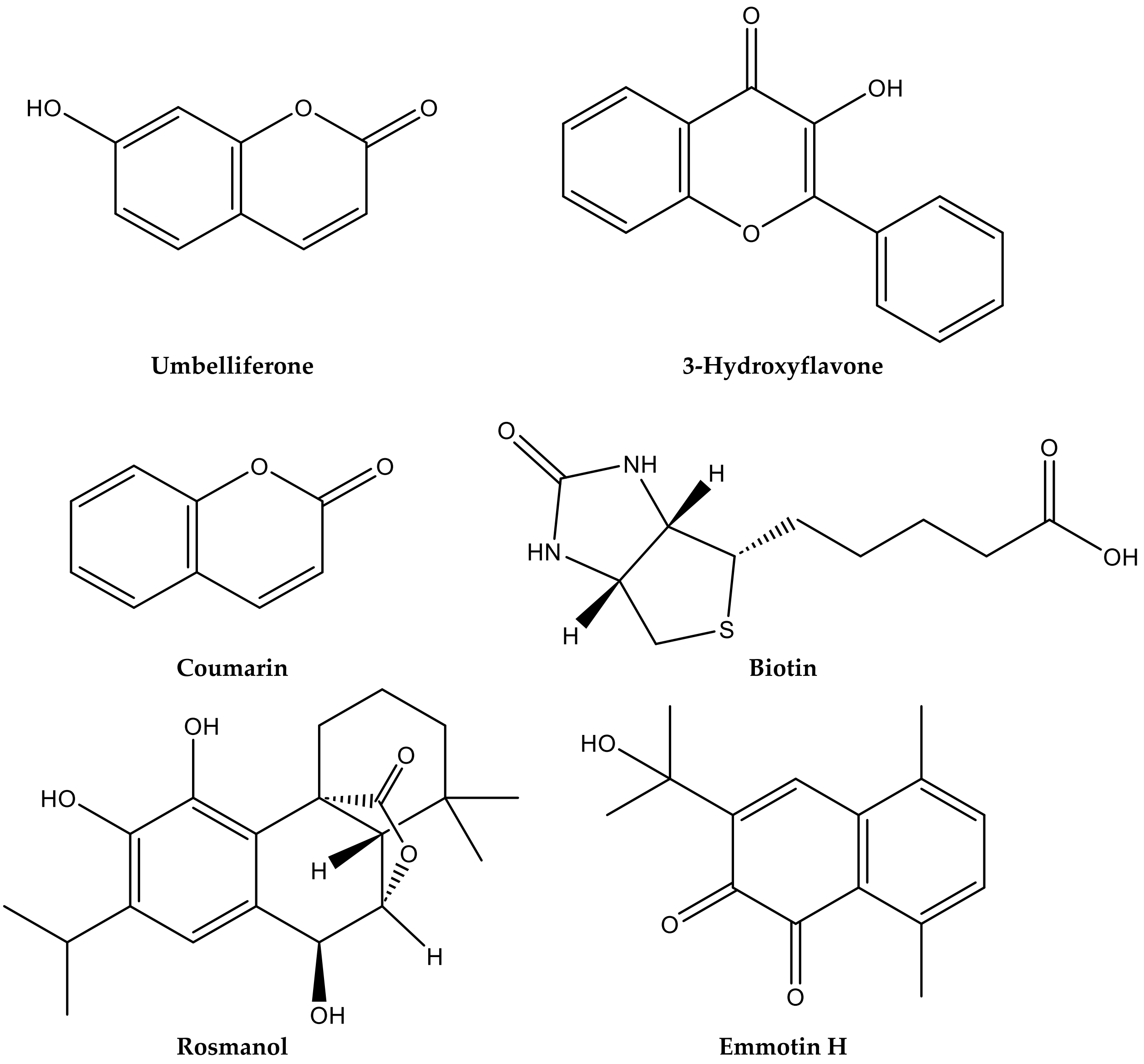

Figure S4. Structures of the majority of compounds identified in extract E (0) of *A. graveolens*.

Table S1. Gradient mobile phase elution

Time (min)	*Eluent A (%)*	*Eluent B (%)*
0	98	2
20	70	30
25	5	95
26	98	2
30	98	2
